# Latent Profiles of Codex-Mapped Additive Functional Classes and Their Association with Nutri-Score in a Sampled Turkish Retail Food Inventory

**DOI:** 10.3390/foods15213838

**Published:** 2026-10-27

**Authors:** Sena Roberts, Muhammet Ali Çakır

**Affiliations:** 1 Department of Nutrition and Dietetics, Faculty of Health Sciences, Acibadem Mehmet Ali Aydinlar University, Ataşehir, 34752 Istanbul, Turkey; 2 Department of Nutrition and Dietetics, Faculty of Health Sciences, Kirklareli University, 39100 Kirklareli, Turkey; cakir34@gmail.com

**Keywords:** food additives, international numbering system, functional classes, codex alimentarius, latent class analysis, nutri-score, nutrient profiling, food labeling, packaged foods

## Abstract

Food additives recur in combinations across packaged foods, but their association with nutritional quality is uncertain. We identified latent profiles of Codex-mapped functional classes from label-declared additives in a sampled Turkish retail inventory and related them to Nutri-Score grade. Of the 6515 products, 3383 with a retained functional-class indicator entered latent class analysis. Five profiles were retained using a prespecified multi-criterion procedure without numerical cutoffs; the fifth, gas-related profile was mapping-sensitive and not interpreted. Investigator-computed grades (2023 algorithm) were analyzed in 2942 products across 56 food subgroups with two-step ordinal models. The raising and texture profile was associated with poorer grades across the scale (odds ratio, 1.99; 95% confidence interval, 1.51–2.63); the oxidation and color-retention profile was inversely associated at the grade-E boundary (0.49, 0.37–0.63); and the coating and structure profile was displaced from the favorable end (imprecise estimate). In profile-only models, these directions held under a structurally reduced specification and after restoring nonsweetener classes of excluded sweeteners and polyols; magnitudes and cutpoint-specific forms, particularly for the raising and texture profile, were specification-sensitive. The associations are subgroup-conditional descriptive contrasts in a sampled, non-national inventory; the profiles describe Codex-mapped declarations with mapping-induced dependence, not verified functions, additive intake, or health effects.

## 1. Introduction

Food additives are among the most extensively shared features of packaged food supplies. In large label databases, >50% of products on the French market carried at least one additive [1], and additives were present in >75% of packaged products across five Latin American countries [2]. Their declarations reveal a recurring co-occurrence structure. Multivariate analyses identified six clusters on the French market [1], eight in Hong Kong [3], and five patterns of additive categories in Brazil [4]. Furthermore, functional class co-occurrence has been documented: among 1118 Brazilian products targeted at infants and children, functional classes frequently co-occur [5].

The structure of these combinations has gained significance beyond description. In the Siga framework, the number and nature of ultra-processing markers contribute to processing classification [6]; cohort work indicates that additives are encountered as mixtures rather than singly [7]. When combinations carry information not captured by counts, then supply-side monitoring may benefit from methods that describe which Codex-linked functional classes are represented together and how many.

Existing evidence on the association between additive patterns and the nutritional quality of the same products is predominantly indirect. Nutri-Score and NOVA show overall alignment while capturing distinct food-level health dimensions [8]. A data-driven clustering of Uruguayan packaged products revealed that additive disclosure inconsistently aligned with nutrient composition [9]. Moreover, additive declarations have served as classification markers: in dietary data from Chilean children, adding ingredient markers, functional-class names, and individual cosmetic additives to food descriptions increased the estimated caloric share of ultra-processed foods from 47.4% to 58.4% [10]. Within one processing category, nutritional quality is itself heterogeneous: of the 13,025 ultra-processed products on the Italian market, 64.3% were graded D or E under the Nutri-Score, and unfavorable profiles were not confined to the subgroups reportedly associated with an elevated noncommunicable disease risk [11]. These studies operate at the level of broad processing categories, treating the declaration as a binary marker, or cluster additives and nutrients jointly. However, how patterns of Codex-mapped functional classes relate to a nutrient profile measure when the two are kept analytically separate remains unexplored. Reporting at the additive level also ties the findings to national authorization lists, whereas the functional classes listed in the Codex class-name system [12] offer an internationally standardized reference for the technological roles listed for declared additives. Codex functional classes and nutrient profile scores are related but non-equivalent constructs. A functional class describes a technological purpose listed for a declared additive; it does not describe the amount used, the function actually performed, dietary exposure, or a processing classification. The two measures are therefore not expected to reflect a common underlying dimension. This distinction is consistent with recent food-contact-material research showing that chemical occurrence or extractability does not necessarily translate into migration or migration-based exposure [13]. The rationale for examining their association is that technological-function requirements and nutrient composition may represent different aspects of the same formulation architecture, so that recurring combinations of declared functions could accompany characteristic nutrient profiles without either measure determining the other.

Turkish label studies have investigated sodium-containing additives [14], phosphorus-based additives [15], and the nutritional profile of ultra-processed and child-targeted products [16,17]. These studies have mainly reported prevalence, whether of an additive class or of a nutritional outcome. However, evidence on how Codex-mapped functional classes are represented together within individual products and on how such combinations relate to the nutrient profile quality of the same products remains scarce.

Therefore, this study aimed to (1) identify latent profiles from 26 Codex-mapped functional-class indicators derived from additive declarations on packaged-food labels in the sampled Turkish retail inventory, and (2) estimate the association between these profiles and Nutri-Score grade, computed by the investigators under the updated 2023 algorithm [18]. A prespecified reducibility analysis investigated whether profile associations were reducible to the overall number of functional classes represented per product.

## 2. Materials and Methods

### 2.1. Study Design and Reporting

This was a cross-sectional analysis of label data from a sampled retail inventory of packaged foods and non-alcoholic beverages in Turkey. No human participants were involved; no ethical approval was required. Reporting followed the STROBE statement for cross-sectional studies [19] alongside the STROBE-nut extension where applicable [20]. The latent class component followed published methodological and reporting guidelines [21,22].

The analysis plan was finalized in a time-stamped internal protocol, not publicly preregistered, before the joint distribution of latent profile membership and Nutri-Score grade was inspected. The number of latent classes, the structural estimator, integration rule, variance procedure, outcome model and its fallback specification, and prespecified sensitivity analyses were all fixed at that point. Departures from the protocol are identified as such in Section 2.7.5.

### 2.2. Data Source and Sampling Frame

Between June 2025 and January 2026, label data were collected from packaged foods and non-alcoholic beverages sold by nine retail chains in Turkey. Physical collection covered 20 outlets in Kırklareli and Istanbul. Retail chains and outlets were selected non-probabilistically based on fieldwork feasibility and product availability. The resulting dataset therefore represents a non-probability sampled retail inventory. Market-share data for the sampled chains and platforms were not available to the investigators, so neither national nor retailer market coverage could be quantified. Three online retail platforms were screened over the same period, both to identify eligible products not encountered in store and to validate or complete label information.

Both investigators conducted the fieldwork. Each outlet was screened category by category; every eligible packaged product on the shelf at the time of the visit was photographed. Product categories organized fieldwork and image files; they were not sampling quotas. The ingredient list, nutrition declaration, and remaining label fields were transcribed from the images into the study database; subsequently, the second investigator examined each transcribed field against the corresponding package image. Independent double-checking of extracted label data has been applied in a comparable packaged-food study [23]. Ambiguous, incomplete, or internally inconsistent declarations were flagged and re-investigated against the image, and where that did not settle the question, against the official manufacturer or retailer product page. When the image and an online source disagreed, the image took precedence. Database quality control aligned with methodological recommendations for comparable in-store census food-label databases [24], with discrepancies corrected where the source information allowed. Records that could not be resolved against a label source retained a quality-control flag; subsequently, analytical eligibility was identified according to the rules in Section 2.6.

**Figure 1:**
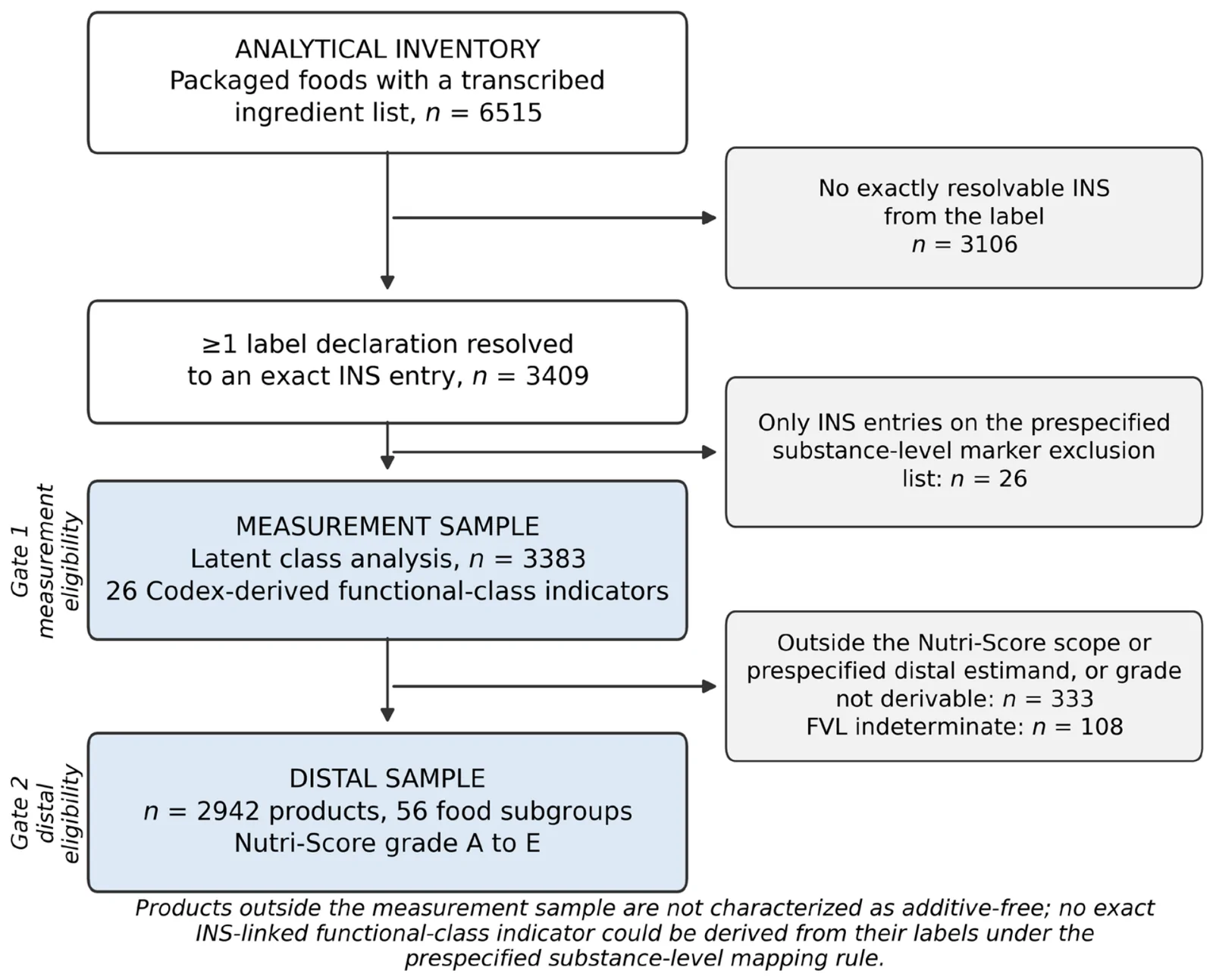
Sample flow through the two eligibility gates. The analytical inventory encompasses 6515 retail packaged foods for which the label ingredient list is transcribed. Gate 1 (measurement eligibility): 3409 products had at least one label declaration resolved to an exact International Numbering System (INS) entry; 26 of these were excluded because all of their exactly resolved entries belonged to the prespecified substance-level marker exclusion list and yielded no retained functional-class indicator, leaving 3383 products for latent class analysis on 26 Codex-derived functional-class indicators. Gate 2 (distal eligibility): 333 products outside the Nutri-Score scope or the prespecified distal estimand, or without a derivable grade, and 108 products with indeterminate fruit, vegetable, and legume (FVL) content were excluded, leaving 2942 products in 56 food subgroups for the distal analyses. The eligibility criteria are provided in Section 2.6. Products outside the measurement sample are not characterized as additive-free; no exact INS-linked functional-class indicator could be derived from their labels under the prespecified substance-level mapping rule. Arrows indicate the flow of products through the two gates, the boxes on the right list the products excluded at each step, and the shaded boxes mark the two analytical samples.

The analytical unit was the label-defined product formulation rather than the barcode or individual package. Barcodes were used during data entry to assist record linkage but were not retained as an analytical database field. Of the 6526 completed records, two confirmed same-source duplicate entries and nine records without transcribed ingredient lists were excluded, yielding an analytical inventory of 6515 products. The database rule was to retain one record per formulation. Pack sizes and package variants carrying the same formulation, and repeated appearances of the same formulation across outlets or online listings, were entered as one record. Records that matched by product identity but differed in their declared ingredient lists were retained as separate observed formulations; a later observation did not replace an earlier one, and such differences were not necessarily interpreted as temporal reformulations. During revision, potentially overlapping records were further screened using normalized brand and product-identification information, standardized declared ingredient lists, and package-image review by both investigators. This review did not identify additional confirmed redundant records requiring exclusion. Because systematic barcode-by-date linkage was not retained, the inventory could not identify every temporal reformulation or establish that the latest formulation of each marketed product had been captured. It should therefore not be interpreted as a synchronized snapshot of the retail food supply. The cleaned analytical inventory and the subsequent eligibility steps are described in Section 2.6 and summarized in Figure 1.

### 2.3. Ingredient Standardization and Additive Identification

Ingredient names were transferred verbatim from the label and subsequently standardized against a controlled ingredient dictionary. Standardization corrected spelling and optical character recognition errors and merged orthographic variants. Compositional meaning-carrying distinctions were preserved, including those between palm, palm kernel, and palm olein oils or between butter and milk fat. Compound ingredient declarations were retained in their label form; their parenthetical components were expanded into separate records to ensure that every declared additive in a compound ingredient entered the analysis.

An additive was assigned a specific International Numbering System (INS) entry only when the label named a substance that unambiguously corresponded to that entry. Assignment used the Turkish Food Codex Regulation on Food Additives [25] for the national regulatory name and the Codex Alimentarius class names and INS for food additives (CXG 36) for the INS entry and its listed functional classes. Declarations that named only a functional class, such as “preservative” or “color”, were recorded as such and were not assigned an INS entry. Flavorings were treated as a separate ingredient category and not as food additives, consistent with the regulatory distinction between a flavoring and a flavor enhancer. A flavor enhancer was coded as an additive only when the substance itself was named. Precautionary allergen statements were separately stored and never treated as ingredient use.

Of the 16,058 ingredient records categorized as additives in the dictionary, 14,197 (88.4%) resolved to a specific INS entry and 1861 (11.6%) did not. The unresolved records were divided into two regulatory classes. In 1623 records (10.1%, 801 products), the label text named only a functional class; however, the Turkish Food Codex Regulation on Food Labeling and Informing Consumers [26] required the functional class alongside the specific name or the E number. In 238 records (1.5%, 229 products), the category name was the legally sufficient declaration, all of them modified starch, for which no E number was required and from which no specific INS entry could be derived from the label. The second class was a consequence of the labeling framework rather than a data gap. The distribution of both classes by food group and by profile is reported in Supplementary Table S12, Panels B and G. Class-name-only declarations were not converted into indicators in the primary analysis because every indicator was defined from an exactly resolved substance and its Codex record. A class name identifies one declared function but not the additive, so the other Codex classes of the same substance cannot be assigned; admitting class names would also combine substance-derived and wording-derived indicators in one measurement vector. Their influence was assessed directly (Section 3.6 and Section 3.8).

### 2.4. Codex-Derived Functional-Class Indicators

Each resolved INS entry was mapped to the functional classes listed in CXG 36. The mapping file was frozen before analysis against one archived revision of the guideline [12]. Codex states that the technological purposes it lists are indicative rather than exhaustive; therefore, the resulting variables describe Codex-listed functional classes for the declared additives and not the functions actually performed in the product.

Two rules in the frozen mapping affect interpretation and are stated here. First, where an INS entry had subdivisions, the entry received the common functional-class set when all subdivision lists were identical (23 codes), and the intersection of the subdivision lists otherwise (16 codes, each carrying a review flag). Second, using a frozen marker exclusion list, sweeteners and polyols were removed before indicator construction. This was an analytical decision rather than a property of the algorithm. For nonnutritive sweeteners, retention would have placed a component of the beverage outcome definition directly inside the measurement model because the updated algorithm assigns them an unfavorable component [18]; to limit related construct overlap, polyols were removed under the same frozen rule because they may substitute for sugars, which the algorithm scores. The exclusion operates at the substance level rather than at the class level: an INS entry on the frozen marker list contributed no indicator at all, including any functional class other than a sweetener listed in CXG 36. Therefore, 26 products whose sole exactly resolvable declarations were entries on this list were left without any retained functional-class indicator and could not enter the measurement model (Figure 1). In 13 out of the 26 products, the sole declaration was steviol glycosides (INS 960), for which sweetener was the only functional class listed; therefore, relaxing the substance-level exclusion would still not yield a retained nonsweetener indicator. In the remaining 13 products, at least one excluded entry was a polyol or sucralose for which the frozen mapping also listed one or more retained nonsweetener classes, including, depending on the substance, bulking agent, humectant, stabilizer, thickener, or flavor enhancer. For these products, the absence of an indicator followed from the substance-level rule rather than from the label. The polyol component of this rule was investigated in a protocol-amendment sensitivity analysis, and the substance-level rule as a whole in a revision-stage specification that restored every nonsweetener class of the marker substances (Section 2.7.5).

CXG 36 lists 27 functional classes. As the marker substances were excluded before functional-class mapping, no sweetener indicator was constructed, leaving 26 Codex-derived functional-class indicators at the product level; the indicators are named individually, with their prevalences, in Supplementary Table S3. When at least one resolved INS entry declared on the label was mapped to that functional class in the frozen mapping, an indicator took the value 1. The number of represented functional classes per product, used as the count covariate in Section 2.7.3, represents the row sum of these 26 indicators. As one INS entry may be assigned to more than one functional class in CXG 36, the resulting binary indicators were not necessarily independent representations of distinct formulation features. Therefore, deterministic and near-deterministic associations among them were separately quantified, distinguishing those forced by the mapping from those originating through product-level co-declaration. The results are presented in Supplementary Table S7, and their consequences for the measurement model are investigated in the sensitivity analyses described in Section 2.7.5.

### 2.5. Nutri-Score

Using the updated 2023 algorithm, the investigators computed the Nutri-Score from label-derived nutrition and ingredient information, with point allocations as given in the scientific update [18] and the category provisions of the French implementing text [27]. It is applied here as a nutrient profile model for research purposes. The Nutri-Score is a voluntary front-of-pack scheme adopted in a small number of European countries. As of 2024, no specific legal regulation for front-of-pack labeling schemes existed in Turkey [28], and no product in this inventory carried the label.

The algorithm was applied in its category-specific form, along with the general algorithm; the algorithm for fats, oils, nuts and seeds; the beverage algorithm; the protein-point rule for cheese; and the protein cap for red meat. Points were derived from energy, saturated fat, sugars, and salt on the unfavorable side and from protein, fiber, and the fruit, vegetable, and legume (FVL) component on the favorable side, per 100 g or 100 mL as sold. Under the general algorithm, nuts and oils are not among the ingredients qualifying for the FVL component [18]; within the fats, oils, nuts and seeds category, oils derived from qualifying FVL ingredients, including olive and avocado oils, were counted [27]. Every product with a non-zero FVL entry was audited against this provision at the ingredient level, and the sensitivity of the results to the audit is reported in Section 3.7. Where fiber was not declared, it contributed zero points under the prespecified label-based derivation rule. Under the Turkish labeling regulation [26], fiber declaration is voluntary; the absence of a declaration is a property of the label and does not imply that the product contains no fiber. The rule was fixed in the analysis protocol, and its influence is investigated in Section 3.7.

Products for which the algorithm does not apply were classified as outside the Nutri-Score scope; when the required label inputs could not be established, a grade was treated as nonderivable. Both conditions are applied as distal eligibility criteria in Section 2.6. Within this study, the market scope of the algorithmically derived information is therefore the sampled retail inventory of packaged foods and non-alcoholic beverages, and grades exist only for the subset of products to which the updated algorithm applies and for which a grade could be derived from the label. These grades are intended for research-based comparison within this sampled inventory. The profile–grade associations were estimated among the 2942 products that met both the measurement and distal eligibility criteria (Section 2.6), and no consumer, socioeconomic, price-positioning, brand-positioning, or retail-channel market segmentation was undertaken.

### 2.6. Eligibility and Exclusions

Eligibility was defined at three levels: the analytical inventory, measurement model, and distal model. The full flow, with the count removed at each step, is depicted in Figure 1.

A product was eligible for the analytical inventory when it was a packaged food or non-alcoholic beverage provided for retail sale in Turkey during the collection period and its label ingredient list had been transcribed into the database (Section 2.2). Unpackaged and loose foods, as well as products sold only in food service, were not recorded. Alcoholic beverages were not represented in the classification used and did not enter the inventory. Nutri-Score derivability was not an inventory criterion and was assessed only at the distal gate. The analytical inventory comprised 6515 products.

Measurement eligibility, which requires information that the latent class model can use, represents the first analytical gate. A product was eligible when at least one label declaration resolved to an exact INS entry that retained a functional-class indicator following the prespecified substance-level marker exclusion (Section 2.4); the intermediate counts are illustrated in Figure 1. This left a measurement sample of 3383 products in 12 food groups and 67 subgroups. Two groups of products were deliberately retained at this gate: 773 out of the 801 products carrying at least one declaration that named only a functional class and 220 out of the 229 products carrying a modified starch declaration (Section 2.3). These products were kept whenever at least one exact retained INS entry was also declared because the unresolved part of such a label reduces the information available for a product but does not make the product ineligible. Products outside the measurement sample were not characterized as additive-free; under the prespecified mapping procedure, no exact INS-linked functional-class indicator could be derived from their labels.

Distal eligibility, which necessitates an applicable and derivable Nutri-Score grade, represents the second gate. Some products, including foods for infants and young children, sports foods, and food supplements, fell outside the scope of the algorithm. For others, a grade could not be derived from the declared information, either because a required input was missing or implausible, because no nutrition declaration was present or because the product was a single-ingredient food exempted from declaring one. Another group had an indeterminate FVL content; their grades were computable under a zero-FVL assumption but were sensitive to it; thus, they were excluded from the primary distal sample and separately investigated. Plain water was excluded on a separate ground. Its grade follows a category rule rather than nutrient composition; therefore, it lies outside the estimand, which concerns the association between the declared latent profile and a composition-derived grade. A sensitivity analysis that retained these products was conducted; its outputs are held by the authors and are available on reasonable request. The counts are shown in Figure 1. As scope under a nutrient profile algorithm and eligibility for the measurement model are separate criteria, products excluded here remained in the measurement model. The distal sample comprised 2942 products in 11 food groups and 56 subgroups, with a median subgroup size of 35 products and the largest subgroup size of 324.

No product was removed from the analytical inventory at either gate. Eligibility was recorded as a set of flags, and every excluded product was retained with its exclusion reason in an exclusion log held by the authors; therefore, both analytical samples could be reconstructed from the analytical inventory.

### 2.7. Statistical Analysis

#### 2.7.1. Measurement Model

Latent class analysis was applied to the 26 binary functional class indicators in the measurement sample (*n* = 3383) using the standard local-independence specification within class and a class prior common to all food subgroups. The local-independence assumption warrants particular attention as the mapping induced part of the dependence among indicators (Section 2.4); this was investigated through the alternative measurement specification and targeted redundancy analysis in Section 2.7.5. Nutrient variables and the Nutri-Score were kept out of the measurement model so that the outcome could not inform profile formation [29]. Solutions from one to twelve classes were estimated using expectation maximization with random starts. Class enumeration followed the prespecified multi-criterion rule rather than any single index: information criteria, bootstrap likelihood ratio test, number of boundary conditional probabilities, replication of the best log-likelihood across starts, half-sample stability under mutual nearest-neighbor matching, and substantive distinguishability of the class profiles. No numerical threshold was prespecified for any of these criteria; the number of classes was chosen by weighing them jointly (Section 3.3). Entropy was descriptively reported rather than as a selection criterion. The enumeration diagnostics and selected solution are reported in Section 3.3.

The profile membership distribution across food subgroups was described by three summaries in the distal sample, all computed under modal assignment and descriptive. Cramér’s V was reported for the subgroup by profile cross-classification. For each profile, membership was coded as a binary indicator, and its total sum of squares decomposed in a one-way classification by food subgroup; the within-subgroup share was computed as one minus the between-subgroup share of that total. The effective number of subgroups for a profile represented the exponential of the Shannon entropy of the distribution of its products across subgroups to ensure that a profile evenly spread across m subgroups has an effective number of m.

The five profiles were named from the functional classes with the highest conditional probabilities within each class. These names summarize dominant patterns of Codex-mapped functional-class indicators; they are not product-category assignments and should not be read as functions verified in the individual products or as validated or health-relevant categories.

#### 2.7.2. Structural Estimator

Latent profile membership was associated with the Nutri-Score grade with a fixed-parameter two-step estimator: the class-conditional indicator probabilities were estimated in the first step and held fixed, while the class proportions and the structural model were estimated in the second [30,31]. The prespecified performance hierarchy did not differentiate between this estimator and maximum-likelihood three-step estimation; the two-step estimator was selected before any profile–grade association was inspected because it estimates the structural model directly, without an intermediate classification–error matrix.

#### 2.7.3. Outcome Model

The five-level ordinal Nutri-Score grade, modeled with a cumulative logit link and a food-subgroup random intercept, constituted the outcome. Food subgroups entered the model as defined in the source classification and were not merged. Fourteen subgroups held fewer than ten products, together containing 44 out of the 2942 products in the distal sample; the random-intercept model partially pooled subgroup intercepts toward the population mean, whereas the fixed-effects specification (Section 3.8) provided a no-pooling sensitivity check. A nine-point adaptive Gauss–Hermite quadrature was employed for evaluating the random-effect integral. The proportional odds assumption was tested globally and profile by profile, with Holm adjustment applied to the profile-specific tests. A fallback was prespecified: when the assumption failed, a partial proportional odds specification [32,33] would be fitted, freeing the violating profile terms across cutpoints and constraining the remaining profile terms to be proportional. The test outcome, the resulting specification, and its comparison with the proportional odds and fully nonproportional models are reported in Section 3.4. Model A contained the profile terms and the food-subgroup random intercept. Model B additionally encompassed the number of represented functional classes as a restricted cubic spline with three knots. As this count was the row sum of the same 26 indicators that defined the profiles, it represented an aggregate summary of the indicator vector rather than an independent confounder; Model B was reported as a reducibility test. The knots were placed at the tenth, fiftieth, and ninetieth percentiles of the functional-class count distribution in the distal sample [34], which gave boundary knots at 1 and 14 and an interior knot at 8. Under the prespecified form, the spline coefficients were held constant across cutpoints.

Effects were reported as odds ratios (ORs) with 95% confidence intervals (CIs) relative to the sparse profile, with an OR >1 indicating higher odds of poorer grades. Absolute-scale results were reported as marginal-grade probabilities, integrated over the estimated random-intercept distribution using an 80-point Gauss–Hermite quadrature. These probabilities were derived from the adopted partial proportional odds Model A, integrated over the estimated food-subgroup random-intercept distribution; the proportional odds form, fixed as the estimand model before the assumption was tested, is reported in the Supplementary Materials for comparison. As the research question concerned the inventory rather than a single subgroup, the marginal estimand was fixed in advance. Conditional probabilities at zero random effect, which represent the model prediction at the center of the estimated random-intercept distribution rather than the inventory-wide marginal distribution, were derived from the proportional odds form. CIs were obtained from 4000 parametric draws using the percentile method; the marginal probabilities from both models and the proportional odds conditional probabilities are reported in Supplementary Table S6.

#### 2.7.4. Variance Estimation

Primary standard errors denote conditional model-based standard errors from the observed information matrix. These do not by themselves propagate the sampling uncertainty of the first-step measurement parameters; therefore, standard errors incorporating that uncertainty were also computed by drawing the first-step parameters from their estimated asymptotic distribution and re-estimating the second-step model with each drawn set of class-conditional indicator probabilities [35], with 100 draws and 21 boundary conditional probabilities held fixed rather than drawn; because these boundary parameters were not drawn, the corrected standard errors may understate measurement uncertainty at the most strongly separated cells. Bootstrap variance procedures were not used for the primary inference because internal calibration checks at the resampling depths feasible here did not show satisfactory inferential calibration; the calibration outputs are retained by the authors. A result whose inferential decision differed between the two variance methods was labeled as variance-method sensitive, and both results were reported. Dependence among products of the same brand was assessed in a revision-stage brand-level cluster bootstrap (Section 2.7.5).

#### 2.7.5. Sensitivity Analyses

Besides the prespecified outcome-definition checks described in Section 2.5, four prespecified model-specification sensitivity analyses were conducted. Food subgroup was entered as 56 fixed effects instead of a random intercept. To address the largest residual local dependencies, the structural model was re-estimated on an alternative measurement solution that frees four direct effects between indicator pairs. No first-step covariance was derived for that parameterization; therefore, it was reported with conditional variance only, and this was stated as a study limitation. The analysis was repeated on the 37 subgroups in which at least two profiles held at least 10% of the products (2308 products, 78.5% of the distal sample), a restriction defined before results were observed to examine whether the profile coefficients rested on within-subgroup contrast. Finally, the count term in Model B was freed across cutpoints as a specification check. A coefficient whose decision changes under a sensitivity analysis was labeled accordingly, and both results were reported.

Three further sensitivity analyses were added following completion of the primary analyses, in response to an internal pre-submission review. They were specified in a dated protocol amendment before they were executed and are described as such rather than as prespecified. They comprised the exclusion of products carrying class-name-only additive declarations, a measurement specification in which polyols were re-included with their nonsweetener Codex classes while keeping the measurement sample fixed to ensure that the 26 marker-only products were not re-admitted, and four- and six-profile measurement solutions. A fourth protocol-amendment sensitivity analysis addressed structural redundancy among the gas-related indicators, a different property from the residual local dependence targeted by the above-mentioned alternative measurement specification. Propellant, packaging gas, and carbonating agent were aggregated into one gas-related composite indicator, reducing the measurement vector from 26 to 24 indicators. The five-class model was re-estimated under the same estimation settings, with the resulting profiles matched to the primary solution using the Hungarian assignment. Class enumeration was repeated on the reduced indicator set under the same diagnostics. The results are reported in Supplementary Table S8. All results are reported in the Supplementary Materials irrespective of outcome.

The following four post-primary robustness analyses were specified after a methodological review of the primary results: incorporation of explicitly declared functional classes from unresolved declarations into the indicators, restoration of the nonsweetener Codex classes of excluded marker substances within the primary measurement sample, repetition of the distal analysis without the products scored under the beverage branch of the algorithm, and a direct re-estimation of the favorable-end contrast within the same fixed-parameter two-step framework under binary and corresponding four-level ordinal outcome specifications. These analyses were not preregistered and were interpreted as post-primary robustness checks. The results of the four post-primary robustness analyses are reported in Supplementary Table S9.

A first set of revision-stage analyses was specified in a dated post-review protocol amendment, written after the reviewer reports were received and before any of these analyses were executed; they are described as revision-stage analyses rather than as prespecified. The amendment defines an outcome-blind structural-reduction rule at the mapping level: two indicators are treated as structurally equivalent when the frozen Codex mapping forces one to imply the other at the code level and their product-level indicator vectors differ for at most one product, and maximal groups of equivalent indicators are merged. Applied to the frozen mapping, the rule merged exactly the propellant, packaging-gas, and carbonating-agent indicators, reproducing the 24-indicator set of the gas-collapse analysis while leaving all one-way containments intact. Class enumeration, both half-sample stability variants, profile matching, and the full distal sequence were repeated on the reduced set, with the number of classes selected by the same multi-criterion record as in the primary analysis. The distal sequence comprised the proportional odds assessment with Holm adjustment, the partial proportional odds model, and the count model, with the count still defined as the row sum of the original 26 indicators. The amendment additionally specified a count-benchmark ladder comparing subgroup-only, count-only, profile-only, and profile-plus-count outcome models; a re-assessment of the first-step variance correction at 100, 500, and 1000 measurement-parameter draws; a cross-tabulation of fiber declaration status by profile and by food group; complete ascertainment tables for the analytical samples; and the regeneration of the observed and model-standardized grade-distribution figure from the partial proportional odds model, with the proportional odds version retained in the Supplementary Materials. No numeric recovery threshold was defined; for each principal contrast, the direction, estimates, confidence intervals, and proportional-odds status under both specifications are reported symmetrically, and all results are reported irrespective of outcome (Supplementary Tables S10–S13).

A second set of revision-stage analyses was specified in a further dated amendment, written after a subsequent reviewer report was received and before these analyses were run; none is described as prespecified. First, products inside and outside the measurement sample were compared descriptively by food group, ingredient number, unresolved declarations, and calculated Nutri-Score grade, without significance tests. Second, in a nonsweetener-function restoration specification, every Codex functional class other than sweetener listed for the excluded marker substances was restored within the measurement sample of 3383 products; the sweetener class itself was not restored. Class enumeration from one to twelve classes, both half-sample stability variants, class selection by the same multi-criterion record without numerical thresholds (recorded in a dated addendum before any distal model was fitted), correlation-based matching to the primary profiles, and the full distal sequence were repeated, with the count defined as the row sum of the restored indicators. A secondary variant additionally admitted the 13 marker-only products that gain an indicator under restoration and was evaluated at the measurement level only. Third, brand names recorded as text at data entry were normalized for case, diacritics, and punctuation and used as clusters; manufacturer and product-family identifiers were not available. The adopted partial proportional odds Model A, with the class-conditional indicator probabilities fixed, was re-estimated in 200 brand-cluster and 200 product-level bootstrap replicates.

### 2.8. Derivation-Chain and Mapping Verification

The derivation chain from the workbook to the indicator matrix was separately audited from the database-level quality control described in Section 2.2. The measurement sample membership rule and the count of unique resolved INS entries were reproduced for all 3383 products. Functional-class assignments inferred from single-INS products and extended using combination constraints from multi-INS products uniquely resolved 99 out of the 186 nonmarker INS codes; the remaining 87 could not be uniquely resolved from the data and were therefore manually verified against CXG 36, code by code, by one author.

Three checks were subsequently applied to the completed mapping. All 87 verified function sets satisfied the constraints the data imposed on them, 86 of which agreed with an independent application of the subdivision rule, the exception being one code for which no subdivision record was comparable. The completed mapping reproduced the indicator rows of 716 products, with no differing cells across 18,616 cells. One code warranted further check: the archived Codex record listed an antifoaming agent alongside its glazing agent, whereas the indicator row of the single product carrying it initially documented only the glazing function. That product did not fall in the audited subsample; therefore, the comparison was extended to the whole measurement sample, where this was the only cell of 87,958 cells at which the frozen mapping and the indicator matrix varied. The product record was rechecked against the label image, and the indicator was set accordingly; therefore, the frozen mapping and the indicator matrix agreed in every cell. For products naming a specific subdivision, the alternative name-level records would have changed four indicator cells in the audit subsample. The frozen mapping is provided in the Supplementary Materials; the constraint definitions, verification records, audit output, and the script that reproduces this chain are retained by the authors and available on reasonable request. In a revision-stage blind re-mapping audit, recorded in a dated addendum to the post-review amendment, the second investigator, blinded to the frozen mapping and to all comparison output, independently re-assigned functional classes to all 87 verified codes from the archived CXG 36-1989 copy, applying the study’s subdivision-aggregation rule to the 14 codes without a directly matching single entry. The independent assignments matched the frozen mapping exactly for all 87 codes and all 262 class assignments.

### 2.9. Software

All analyses were performed in Python 3 (NumPy 2.4.4, SciPy 1.17.1, pandas 3.0.2, scikit-learn 1.8.0, StepMix 3.0.0 [36]). In this study, the adaptive quadrature kernel, two-step estimator, and first-step variance correction were implemented. Independent implementations (StepMix 3.0.0; R 4.3.3 with ordinal 2023.12-4 and VGAM 1.1.9) agreed with the study implementation to a maximum absolute difference of 4.1 × 10^−4^ across the compared log-likelihoods, model parameters, and class-conditional grade probabilities (largest for a fifth-profile parameter of the partial proportional odds model). The custom first-step variance correction was checked by confirming that re-estimation with the first-step estimates left unperturbed reproduced the primary estimates. Random seeds were fixed at 20260822 and 20260824 for the measurement step and calibration study, respectively.

## 3. Results

### 3.1. Analytical Samples and Nutri-Score Distribution

The measurement sample comprised 3383 products in 12 food groups and 67 subgroups; the distal sample encompassed 2942 products in 11 food groups and 56 subgroups (Table 1; Supplementary Table S1). The loss between the measurement and distal samples was 1.7–15.7% across profiles. Compared with the 3132 products outside the measurement sample, measurement-eligible products listed more ingredients (median 13, IQR 8–21, against 2, 1–5), came more often from confectionery, biscuits, and snacks (27.5% against 8.7%) and less often from bakery, cereal, and pasta (13.3% against 25.0%) and from oils, margarines, and fat spreads (1.1% against 5.2%). Among products with a calculated grade, they more often received grades D or E (76.5% against 32.8%). Of the excluded products, 98.1% carried no ingredient record classified as an additive (Supplementary Table S12, Panel H). Measurement eligibility therefore selected multi-ingredient formulations with at least one retained exact INS-linked functional-class indicator, and the associations reported below are conditional on that selection.

Among products meeting both the measurement and distal eligibility criteria, grades D or E accounted for 77.3% (Table 1). This proportion is conditional on the analytical eligibility criteria and is not a prevalence estimate for the Turkish packaged-food supply. Among food groups, the proportion of grade E products ranged from 10.0% in dairy products and milk-based beverages to 76.3% in confectionery, biscuits, and snack foods (Supplementary Table S2).

### 3.2. Functional-Class Indicators

All 26 indicators were represented. Six functional classes occurred in at least 50% of the measurement sample: acidity regulator (67.8%), emulsifier (65.6%), stabilizer (64.0%), sequestrant (60.1%), thickener (57.9%), and antioxidant (50.8%); complete prevalences are listed in Supplementary Table S3.

Mapping-induced dependence was evident: propellant and packaging gas exhibited identical product-level vectors, carbonating agent differed by one product, and 15 out of 17 deterministic implications were forced by mapping at the code level, the remaining two being empirical containments. Of the 325 indicator pairs, 203 exceeded the bootstrap 95th-centile residual threshold; the four largest residuals were freed in the alternative measurement model, whereas the gas-related pairs showed negligible residuals (Supplementary Table S7).

### 3.3. Five Latent Profiles

Information criteria monotonically decreased from one to twelve classes, and the bootstrap likelihood ratio test favored each additional class from three to eight; therefore, none identified an upper bound. Half-sample stability deteriorated beyond five classes: all classes were recovered in 42 out of 50 splits at five classes compared with 27 out of 50 at six, whereas boundary conditional probabilities increased from 21 to 60 at five and eight, respectively. The five-class solution was retained on stability, identification, and interpretability grounds; entropy was 0.980 (Supplementary Table S4). No numerical cutoff for recovery, boundary probabilities, or class size had been prespecified. Between four and five classes, recovery fell from 47 to 42 out of 50 splits, boundary probabilities increased from 9 to 21, and the smallest class fell from 20.7% to 5.2% of products; between five and six classes, recovery fell to 27 out of 50 and boundary probabilities increased to 34. Five classes were retained by weighing these criteria jointly rather than by a threshold; four- and six-class solutions and two further specifications are therefore reported alongside it (Section 3.6 and Section 3.8).

**Figure 2:**
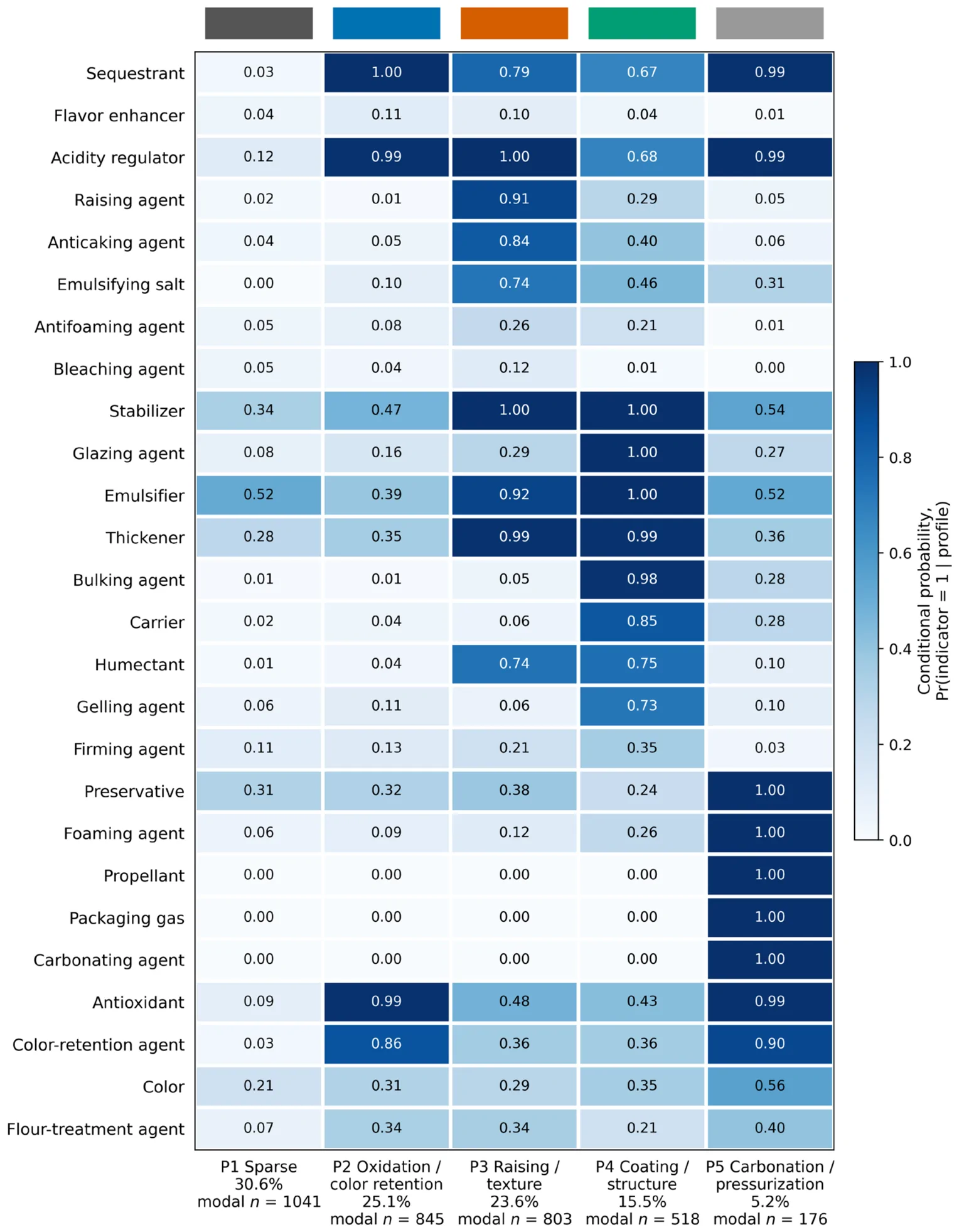
Conditional probabilities of the 26 Codex-derived functional-class indicators across the five latent profiles in the measurement sample (*n* = 3383). Cells exhibit the probability that an indicator is present conditional on profile membership. Profile names summarize dominant function patterns and are not product-category assignments. Column labels show model-estimated profile shares and modal-assignment counts. Rows are ordered by the profile with the highest conditional probability, assessed before rounding. Values are rounded to two decimals; displayed 0.00 and 1.00 values are not necessarily exact boundaries. The colored bars above the columns identify the profiles and match the profile colors used in Figure 3 (P1, dark gray; P2, blue; P3, orange; P4, green; P5, light gray); the color scale on the right gives the conditional probability.

The full conditional-probability matrix is depicted in Figure 2. Profile 1 (sparse) showed no indicator probability reaching 0.70. Profile 2 (oxidation and color retention) was dominated by sequestrant, acidity regulator, antioxidant, and color-retention functions; Profile 3 (raising and texture) by acidity regulation, stabilization, thickening, emulsification, raising, and anticaking; and Profile 4 (coating and structure) by glazing, stabilizing, emulsifying, thickening, bulking, carrier, and gelling functions. Profile 5 (carbonation and pressurization) was dominated by the preservative, propellant, foaming, carbonating, and packaging-gas indicators, all estimated at the boundary value of 1.00. All five functional classes were assigned to carbon dioxide (INS 290) in the frozen mapping; therefore, Profile 5 is reported descriptively and is not substantively interpreted.

Profiles were strongly structured across food subgroups (Cramér’s V 0.684 in the distal sample). For Profiles 1–4, 62%–73% of the membership variation lay within subgroups, and the effective number of subgroups was 10.4–23.1. Profile 5 was more concentrated, with 95.8% of its membership variation lying between subgroups, presence in only eight subgroups, and an effective subgroup number of 4.6.

### 3.4. Profiles and Nutri-Score Grade

The likelihood ratio test (chi-square, 108.69; df = 12; *p* < 0.001) and BIC disfavored the proportional odds model. Profile-specific tests using Holm adjustment identified violations for Profiles 5 (*p* < 0.001), 2 (*p* < 0.001), and 4 (*p* < 0.001) but not for Profile 3 (*p* = 0.060). Therefore, the prespecified partial proportional odds specification was fitted, with the three violating profile terms freed across cutpoints and Profile 3 constrained; it was preferred on both AIC and BIC over the proportional odds and fully nonproportional models, and the fully nonproportional model did not meaningfully improve fit (likelihood ratio chi-square, 4.81; df = 3; *p* = 0.19). The omnibus test of the profile terms was significant (chi-square, 161.18; df = 13; *p* < 0.001). The estimated subgroup standard deviation was 1.694, corresponding to a 0.466 intraclass correlation on the latent scale.

**Figure 3:**
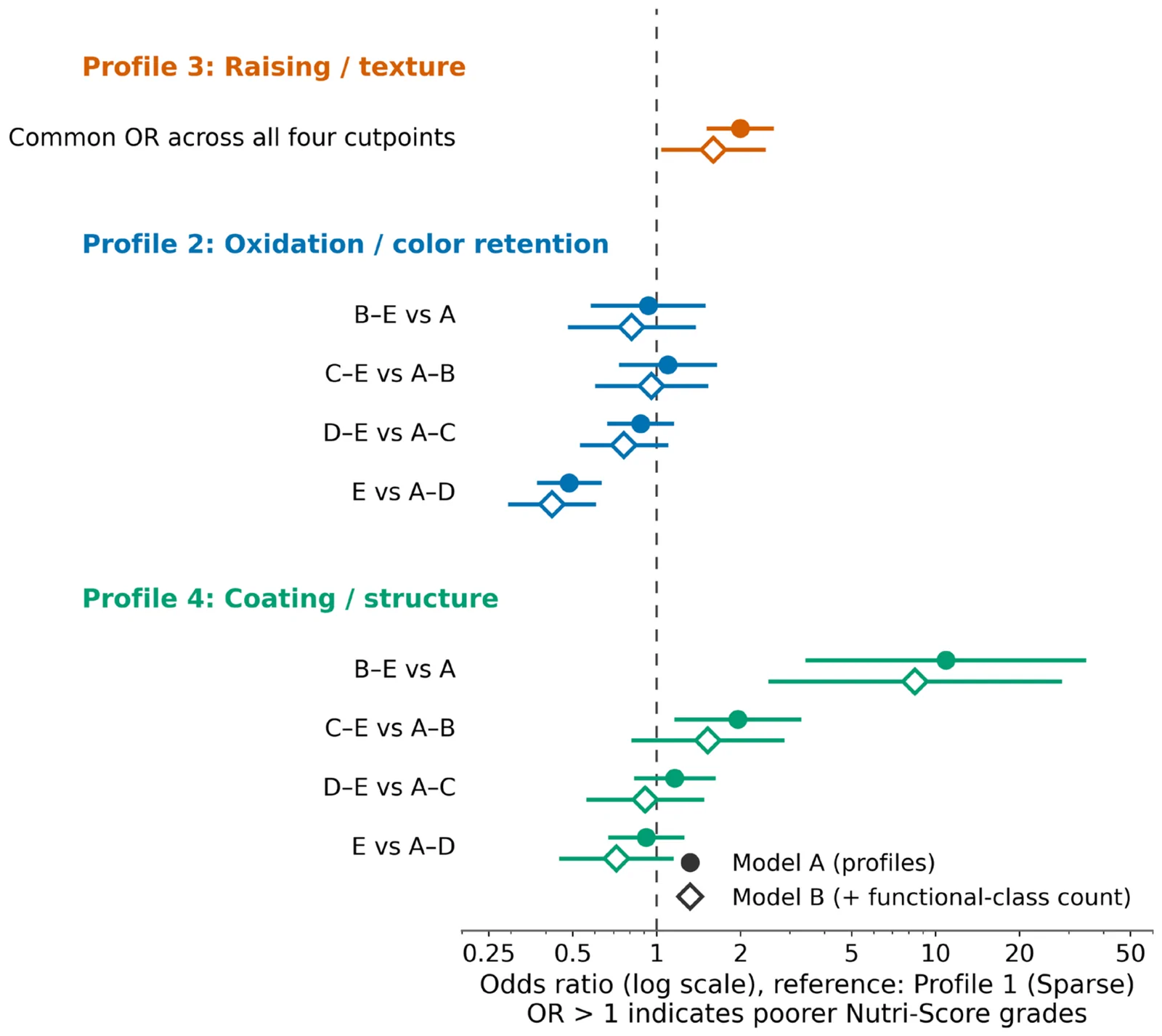
Common and cutpoint-specific associations between latent profiles and the Nutri-Score grade in the distal sample (*n* = 2942 products across 56 food subgroups), expressed as odds ratios (ORs) with 95% confidence intervals (CIs) on a logarithmic scale. Reference profile: P1 Sparse; ORs >1 indicate higher odds of poorer Nutri-Score grades. Model A encompasses the profile terms; Model B further includes functional-class count as a restricted cubic spline. The raising and texture profile term is common across all four cutpoints; the proportional odds assumption is rejected for the remaining three profile terms, which are therefore cutpoint-specific. Both models comprise a food-subgroup random intercept and use fixed-parameter two-step latent-class estimation. Under modal assignment, only 4 out of 447 P4 products are grade A. Colors identify the profiles (blue, Profile 2; orange, Profile 3; green, Profile 4); filled circles show Model A and open diamonds show Model B. The letters A to E in the row labels denote Nutri-Score grades, and each row gives the cutpoint contrast (for example, B–E vs A compares grades B to E with grade A). Profile 5, which is mapping-sensitive and not substantively interpreted, is omitted from this figure; its estimates are shown in Supplementary Figure S1 and Supplementary Table S14.

The ORs relative to Profile 1 are depicted in Figure 3 and summarized in Table 2, with additional post-primary robustness analyses reported in Supplementary Table S9. Because the profile associations differed in both direction and proportional-odds behavior, the results are presented as three distinct patterns rather than as a single ordered gradient.

Profile 3 showed a uniform shift toward poorer grades, with a common OR of 1.99 across all four cutpoints (95% CI, 1.51–2.63; *p* < 0.001).

Profile 2 showed no evidence of association at the first three cutpoints and lower odds of being in grade E versus grades A–D at the fourth (0.49; 95% CI, 0.37–0.63; *p* < 0.001). One common OR for this profile would have been 0.67 and would have averaged over this pattern.

Profile 4 exhibited a contrasting boundary pattern. The OR was 10.87 at the B–E versus A cutpoint (95% CI, 3.41–34.68; *p* < 0.001) and 1.95 at C–E versus A–B (95% CI, 1.16–3.30; *p* = 0.012), with no evidence of association at the two upper cutpoints. Under a proportional odds specification, the common OR for this profile was 1.12 (*p* = 0.412). The B–E versus A estimate rested on sparse observed support, as only 4 out of the 447 modally assigned Profile 4 products received grade A. A direct binary re-estimation dichotomizing grades into A–B and C–E yielded an OR of 1.86 (95% CI, 1.05–3.32) for grades C–E versus A–B, comparable to the same contrast under the primary five-level partial proportional odds model (1.95) and the four-level ordinal sensitivity model (2.30; Supplementary Table S9). Therefore, the much larger B–E versus A estimate is a sparsely supported grade-A boundary contrast and should not be interpreted as the overall magnitude of the Profile 4 association; it is reported for completeness and is not used as a measure of magnitude in the Discussion or Conclusions.

The Profile 5 estimates are reported in Supplementary Table S14 and are not interpreted for the reason provided in Section 3.3.

**Figure 4:**
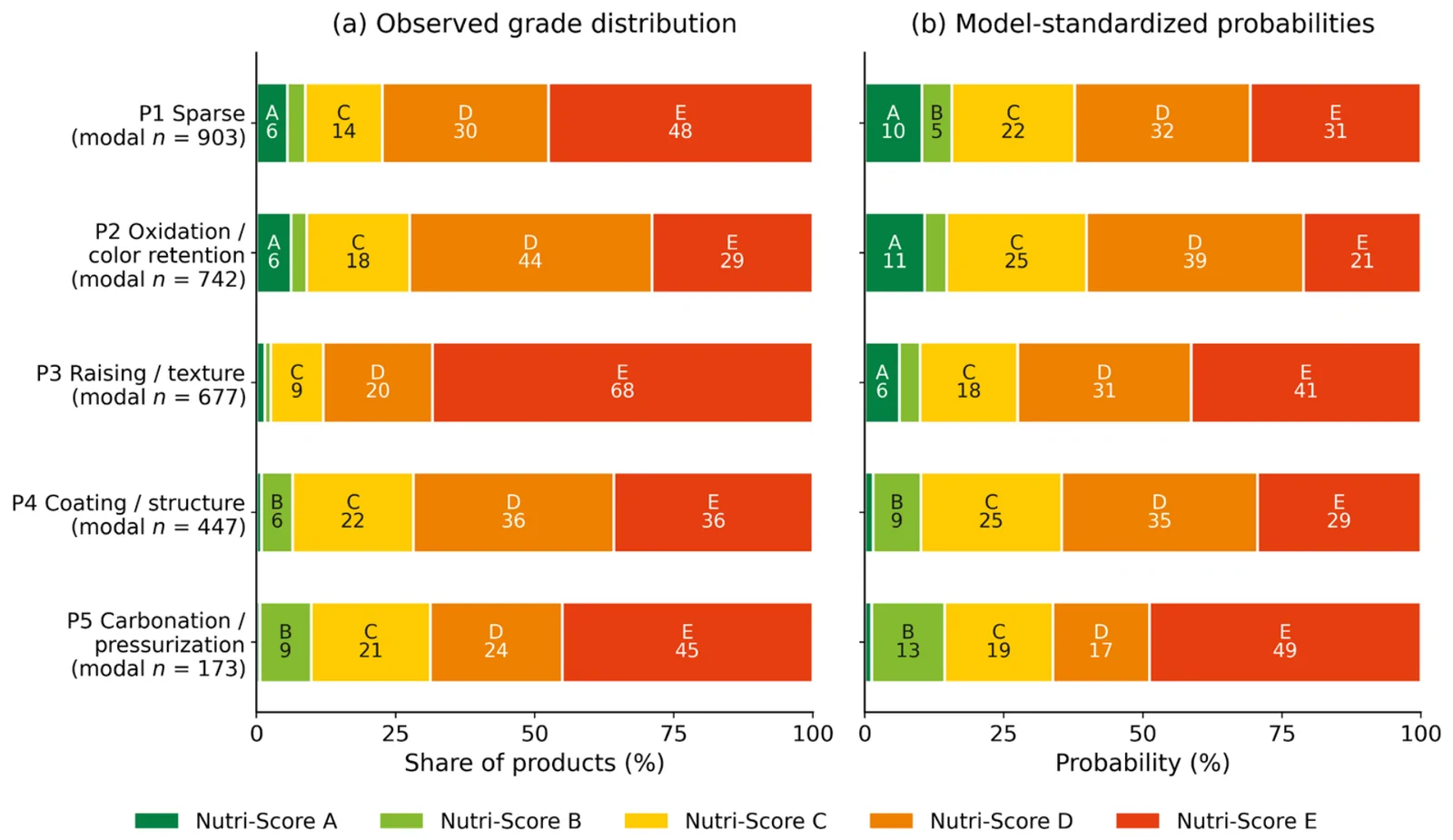
Observed and model-standardized Nutri-Score grade distributions by latent profile in the distal sample (*n* = 2942 products across 56 food subgroups). Panel (**a**) shows the observed grade distribution under modal profile assignment; profile sizes in the axis labels are modal-assignment counts. Panel (**b**) shows model-standardized marginal-grade probabilities from the partial proportional odds model reported in Table 2, integrated over the estimated food-subgroup random-intercept distribution. Panel (**b**) is model-based and should not be interpreted as the observed grade prevalence within each profile. Corresponding 95% CIs are shown in Supplementary Table S6. Displayed percentages are rounded to the nearest whole number. Colors denote Nutri-Score grades A to E, as shown in the legend.

The observed and model-standardized grade distributions are compared in Figure 4. Under modal assignment, the proportion at grade E ranged from 29.0% in Profile 2 to 68.4% in Profile 3, whereas the marginal probabilities from the partial proportional odds model reported in Table 2, integrated over the estimated subgroup distribution, were less dispersed across profiles: grade E ranged from 21.1% to 48.8% and grade A from 1.2% to 10.8%. The proportional odds sensitivity version of this panel is retained in the Supplementary Materials; the largest absolute cell-wise difference between the two versions was 0.156 at grade D of the noninterpreted Profile 5, and within Profiles 1 to 3, the largest difference was 0.065 at grade D of Profile 2. The attenuation of between-profile differences following model standardization aligns with the strong food-subgroup structuring described in Section 3.3. The marginal probabilities should not be interpreted as observed prevalences. Complete distributions with 95% CIs for both the marginal and zero-random-effect conditional estimand are reported in Supplementary Table S6.

### 3.5. Functional-Class Count

Functional-class count varied across profiles, with medians of 2, 7, 11, 12, and 12 in Profiles 1, 2, 3, 4, and 5, respectively. Overlap with the reference profile in count values was correspondingly restricted: Profiles 2–5 shared 6, 5, 4, and 3 distinct count values with Profile 1, respectively.

Adding the functional-class count as a proportional restricted cubic spline did not improve fit (likelihood ratio chi-square, 1.92; df = 2; *p* = 0.38; BIC higher by 14.1). Allowing the count term to differ across cutpoints improved the likelihood (chi-square, 26.86; df = 6; *p* < 0.001) but was not supported by BIC, which was higher by 21.1 than under the proportional specification.

A revision-stage benchmark ladder placed the count and the profiles on the same footing (Supplementary Table S11). Relative to a subgroup-only model, the count alone improved fit modestly (likelihood ratio chi-square, 9.36; df = 2; *p* = 0.009). The profile-only model was preferred over the count-only model by a BIC difference of 64.0; adding the profiles to the count-only model improved fit strongly (chi-square, 153.7; df = 13; *p* < 0.001), whereas adding the count to the profile-only model did not (chi-square, 1.92; df = 2; *p* = 0.38). The profile-only model therefore provided a substantially better descriptive fit than the count-only model. Under the nonsweetener-function restoration specification, in contrast, the count added information beyond the profiles (Section 3.6).

The three principal associations retained their directions with the included count term (Figure 3; Table 2). The common OR for Profile 3 was 1.60 (1.04–2.46), the fourth-cutpoint OR for Profile 2 was 0.42 (0.29–0.60), and the first-cutpoint OR for Profile 4 was 8.44 (2.51–28.37). The adjacent Profile 4 C–E versus A–B estimate was attenuated from 1.95 (1.16–3.30) in Model A to 1.52 (0.81–2.87) in Model B. As the count is an aggregate summary of the same indicators that define the profiles, this comparison represents a reducibility test rather than an adjustment for an independent covariate; the limited overlap in count values further restricts what it can separate.

### 3.6. Sensitivity Analyses

Sensitivity analyses were grouped according to the threat they address and categorized into prespecified, dated protocol-amendment, post-primary, and revision-stage analyses. The prespecified set was defined in the time-stamped internal protocol; the revision-stage set was defined in a dated post-review amendment written during peer review, before execution (Section 2.7.5); none was publicly preregistered. The post-primary checks are presented in Supplementary Table S9.

Measurement-model robustness. Among the prespecified analyses, re-estimating the structural model on the alternative measurement solution with four freed direct effects left 15 out of 16 coefficients with unchanged decisions. The fourth-cutpoint coefficient for Profile 4 changed from an OR of 0.92 (*p* = 0.588) to 0.58 (*p* < 0.001); it was labeled as sensitive to the measurement-model specification, and both results were reported. Standard errors incorporating first-step measurement uncertainty increased the primary standard errors by less than 0.4% and changed no decision. A revision-stage re-assessment at 100, 500, and 1000 measurement-parameter draws left the maximum increase below 0.4% at every step for the proportional odds coefficients; for the adopted partial proportional odds model, the assessment was conducted at 100 draws under the documented computational rule and showed increases of the same order, below 0.4% (Supplementary Table S13). Under the protocol amendments, re-including polyols with their Codex functional classes other than the sweetener and fitting four- and six-profile solutions retained the direction of the three interpreted associations (Supplementary Table S5); the gas-collapse analysis is reported at the end of this section. Two post-primary checks directly addressed the measurement construct. First, direct incorporation of explicitly declared functional classes altered only 33 out of 87,958 product-by-indicator cells, consistent with the declared class being already represented by an exactly resolved entry in most of these products, and under the locked warm-start re-estimation, replicated the primary profile solution (matched modal agreement, 1.000); the principal distal associations were essentially unaffected. Second, restoring the nonsweetener Codex classes of the excluded marker substances within the primary measurement sample attenuated the Profile 3 estimate from 1.99 to 1.69 while retaining its direction and inferential conclusion; the principal Profile 2 and 4 estimates changed slightly. That check held the five-class solution fixed; the revision-stage restoration specification described next repeated class selection.

The revision-stage nonsweetener-function restoration specification (Section 2.7.5) addressed the substance-level marker exclusion directly. Restoring every nonsweetener Codex class of the excluded marker substances changed 617 indicator cells in 320 out of the 3383 products, mostly bulking agent, flavor enhancer, and humectant; because flavor enhancer is the only nonsweetener class listed for several intense sweeteners, the restoration partly reintroduced sweetener use through that indicator (150 cells). Information criteria again declined through twelve classes. Half-sample recovery was complete at three classes, but that solution merged the interpreted profiles. Recovery was low at four and five classes (16 and 19 out of 50 splits in Variant B; 16 and 26 out of 50 half-to-half) and, in Variant B, highest at six classes among solutions with four or more classes (37 out of 50; 29 out of 50 half-to-half), with 31 boundary probabilities against 28 at five classes. At seven classes, Variant B recovery fell to 3 out of 50 and boundary probabilities rose to 52. Six classes were selected by the same multi-criterion record, documented in a dated addendum before any distal model was fitted. The six-class solution recovered a counterpart for each primary profile (matched conditional-probability correlations 0.93 for the sparse reference, 1.00 for oxidation and color retention, 0.98 for raising and texture, 0.87 for coating and structure, and 0.97 for carbonation) and added one intermediate class of 418 products dominated by stabilizer, emulsifier, and thickener, with partial glazing and bulking functions. At five classes, in contrast, the restored solution did not separate the coating and structure profile (matched correlation 0.78): 322 out of its 518 products were assigned to the raising and texture-like class (Supplementary Table S15).

In the distal sequence on the six-class restoration solution, the proportional odds assumption was rejected for every restored profile term, including the raising and texture counterpart (Holm-adjusted *p* = 0.006). The oxidation and color-retention association at the grade-E boundary was retained (OR, 0.45; 95% CI, 0.33–0.60), as was the displacement of the coating and structure profile from the favorable end in the less sparse C–E versus A–B contrast (1.85; 1.09–3.13; sparse B–E vs. A contrast, 6.95; 2.67–18.07). The raising and texture estimates remained above 1 at all four cutpoints (1.14 to 2.08), but only the C–E versus A–B interval excluded 1 (2.08; 1.08–4.02); a single proportional summary would have been 1.41 (1.03–1.95). Unlike the primary specification, the restored count added information beyond the restored profiles (likelihood ratio chi-square, 9.51; df = 2; *p* = 0.009). With the count included, the oxidation and color-retention association retained its direction (0.28; 0.18–0.45); the coating and structure C–E versus A–B contrast was 0.88 (0.44–1.80), and only its sparse B–E versus A contrast remained above 1 (3.32; 1.15–9.61); and the raising and texture estimates were no longer consistent in direction across cutpoints (0.64 to 1.18, all intervals including 1). A secondary variant that also admitted the 13 marker-only products gaining an indicator (*n* = 3396) reproduced the six-class solution (matched correlations 0.87 to 1.00). The marker exclusion therefore shaped the class structure and the strength and form of the raising and texture association, whereas the directions of the three principal associations in the profile-only model were retained.

### 3.7. Outcome-Definition Robustness

Among the prespecified analyses, two rules in the outcome definition were investigated. For all 438 distal products carrying a non-zero entry, the FVL component was audited at the ingredient level; the largest correction the audit can support would change 3 grades out of 2942 and move no coefficient by >0.05 on the log-odds scale. Assigning subgroup median values for undeclared fiber in the 285 products whose food group rendered a non-zero value plausible changed 13 grades and decreased the common OR for the raising and texture profile from 1.99 to 1.98. Excluding those products moved the common OR to 1.64, and excluding all 546 products without a fiber declaration moved it to 1.74. Undeclared fiber was handled algorithmically as zero points rather than interpreted as an observed zero, so the raising and texture association was directionally consistent but magnitude-sensitive to the handling of undeclared fiber. A revision-stage cross-tabulation showed that the absence of a fiber declaration varied modestly across the reference and the three interpreted profiles (15.6% to 23.9% of distal products without a declaration) but markedly across food groups, reaching 97.1% in processed meat and poultry, 90.3% in oils and margarines, and 70.7% in frozen foods (Supplementary Table S12). These checks indicate greater sensitivity to product retention than to the fiber values assigned to undeclared observations. Subsequently, two post-primary checks were performed. The direction of the three principal associations was preserved by excluding all products scored under the Nutri-Score beverage branch. For Profile 3, all four threshold-specific estimates remained >1; however, proportional odds were no longer retained. The direct binary re-estimation of the favorable-end contrast is discussed in Section 3.4. The 108 products excluded from the distal sample because their FVL content could not be determined are characterized by profile and food group in Supplementary Table S12, Panel E; this selection step is distinct from the ingredient-level audit of the 438 products with a non-zero FVL entry.

### 3.8. Structural-Specification Robustness

Replacing the subgroup random intercept with 56 fixed effects changed the coefficients for Profiles 2, 3, and 4 by at most 0.017 on the log-odds scale, and the omnibus profile test remained strongly significant (chi-square, 162.34; df = 13; *p* < 0.001; random-intercept counterpart, 161.18; Supplementary Table S11). Restricting the analysis to the 37 subgroups in which at least two profiles held at least 10% of the products (2308 products, 78.5% of the distal sample) did not change the omnibus decision or the ordering of the profile coefficients. A revision-stage leave-one-broad-food-group-out analysis re-estimated the adopted partial proportional odds model eleven times, each excluding one broad food group: the grade-E boundary estimate for the oxidation and color-retention profile remained inverse in all ten estimable fits (ORs 0.46 to 0.53), and the fit excluding non-alcoholic beverages converged but had a non-positive-definite Hessian and a non-increasing cutpoint sequence, so it is reported as non-estimable (Supplementary Table S10). Under the protocol amendment excluding class-name-only products, the distal analysis was limited to 2269 products with the primary measurement model held fixed; the three interpretable associations persisted, and the estimates were descriptively interpreted because the Hessian of that fit was not positive definite (Supplementary Table S5). In the revision-stage brand-level cluster bootstrap (654 brand clusters in the distal sample; largest cluster, 131 products; 200 replicates per scheme), brand-cluster bootstrap standard errors for the three non-sparse principal terms were 1.23 to 1.36 times the product-level bootstrap standard errors and 1.33 to 1.44 times the model-based standard errors. The brand-cluster percentile intervals excluded 1 for the common raising and texture term (1.47–3.03) and the oxidation and color-retention E versus A–D term (0.33–0.67) but not for the coating and structure C–E versus A–B term (0.99–4.63). For the sparse coating and structure B–E versus A contrast, 6 brand-cluster and 10 product-level replicates gave odds ratios above 1000, consistent with near-separation from its sparse grade-A support, so bootstrap standard errors were not informative for that term. For the three non-sparse terms, brand-level dependence therefore widened the intervals moderately without reversing any direction; no replicate was excluded for non-convergence or invalid cutpoints (Supplementary Table S16).

A fourth protocol-amendment analysis aggregated propellant, packaging gas, and carbonating agent into one gas-related composite indicator. This approach preserved the three substantively interpreted profiles, with matched conditional-probability correlations of 0.995, 0.986, and 0.994 for Profiles 2, 3, and 4, respectively. The raising and texture association remained proportional across cutpoints and increased from 1.99 (95% CI, 1.51–2.63) to 2.27 (1.66–3.10). The coating and structure association at B–E versus A was attenuated from 10.87 (3.41–34.68) to 5.37 (1.87–15.45); the adjacent C–E versus A–B estimate decreased from 1.95 to 1.14 and was not retained after the collapse, whereas the two upper-cutpoint estimates remained null. The oxidation and color-retention association remained inverse; however, the proportional odds assumption was no longer rejected under the same Holm-adjusted procedure (adjusted *p* = 0.10, unadjusted *p* = 0.051; common OR, 0.76; 95% CI, 0.59–0.98). Therefore, its direction was retained, whereas its cutpoint-specific form was not. Profile 5 was not recovered as a corresponding profile (matched correlation 0.374), and its products were redistributed across the alternative solution, whereas the sparse reference profile was divided into two (matched correlation, 0.750). The subgroup variance structure was comparable to that of the primary model (Supplementary Table S8). The class-enumeration trajectory offered additional evidence that Profile 5 was strongly mapping-sensitive: under the 26-indicator specification, the same 176-product pocket generated the smallest class at every solution from five to ten classes, and that set was exactly the products declaring carbon dioxide, whereas it was not recovered once the three gas-related indicators were aggregated (Supplementary Table S8). The principal directional findings for Profiles 2–4 were retained; however, the form and magnitude of some associations changed. Profile 5, in contrast, was sensitive to the representation of the gas-related indicators.

A revision-stage analysis specified in the dated post-review amendment (Section 2.7.5) then treated the reduced indicator set as a specification in its own right rather than as a five-class re-estimation. The outcome-blind reduction rule, defined at the mapping level without reference to the distal results, merged exactly the three gas-related indicators and reproduced the 24-indicator set. Class enumeration was repeated with the number of classes selected by the same multi-criterion record as in the primary analysis. Information criteria again declined monotonically with additional classes, and four classes represented the largest solution with complete half-sample recovery in both matching procedures (50 out of 50 splits in each; three classes were equally stable, but the information criteria still improved markedly from three to four classes, and only the four-class solution recovered the interpreted primary profiles), whereas stability collapsed at five classes (6 out of 50 in Variant B and 8 out of 50 half-to-half) and the boundary-parameter count rose from 5 to 15 (Supplementary Table S10). The four-class solution recovered the sparse reference and the three interpreted profiles nearly exactly, with matched conditional-probability correlations of 1.000 for the reference, 0.995 for oxidation and color retention, 1.000 for raising and texture, and 0.995 for coating and structure, and a nearly diagonal modal cross-classification; of the 176 Profile 5 products, 123 were absorbed into the oxidation and color-retention-like class and 48 into the coating and structure-like class.

The distal sequence was repeated in full on the four-class solution. The proportional odds assumption was again rejected globally (chi-square, 40.9; df = 9; *p* < 0.001), the Holm-adjusted procedure freed only the coating and structure class, and the count term again added no detectable improvement (chi-square, 3.02; df = 2; *p* = 0.22). The raising and texture association was essentially identical under both specifications (common OR, 1.99; 95% CI, 1.51–2.62, against 1.99, 1.51–2.63, in the primary model). The oxidation and color-retention association remained inverse but proportional (common OR, 0.71; 0.57–0.88); its direction was therefore supported under both specifications, whereas its cutpoint-specific form was again not supported. The coating and structure association retained its direction with attenuated magnitude at the grade-A boundary (6.59; 2.45–17.71, against 10.87; 3.41–34.68) and a widened adjacent contrast whose interval included 1 (1.36; 0.85–2.19, against 1.95; 1.16–3.30). Direction, estimates, and proportional-odds status under both specifications are reported for every principal contrast in Table 2 and Supplementary Table S10. Across the two specifications, the direction of the three principal associations was supported in both, whereas magnitude and cutpoint-specific form were specification-dependent; the interpretation in Section 4 is restricted to these direction-level findings. These analyses address the strongest structural redundancy in the indicator set and do not eliminate the local dependence more generally present in the data, which is characterized in Supplementary Table S7.

## 4. Discussion

### 4.1. Principal Findings

Latent class analysis of 26 Codex-mapped functional-class indicators identified five profiles in the sampled Turkish retail inventory, three of which were substantively interpreted, with the sparse profile as reference; their associations with the Nutri-Score grade were estimated after accounting for food subgroup. In the primary specification, these associations did not form a single gradient: the raising and texture profile showed a shift toward poorer grades across the scale, the oxidation and color-retention profile varied at the grade-E boundary, and the coating and structure profile differed at the favorable end (Section 3.4). These patterns were not ordered by functional-class count: the raising and texture and coating and structure profiles had comparable median counts (11 and 12) but were associated with different parts of the grade distribution. When functional-class count was included, the three principal associations retained their directions; however, not every cutpoint-specific estimate retained the same inferential conclusion. As count and profiles were derived from the same indicators and their count distributions only partially overlapped, this comparison represents a reducibility test rather than an adjustment for an independent covariate (Section 3.5).

The partial proportional odds specification mattered for interpretation: under a proportional specification, the coating and structure profile appeared unrelated to the grade (OR, 1.12), whereas the oxidation and color-retention association would have been averaged to a common OR of 0.67. Therefore, a proportional model would have obscured both cutpoint-specific patterns. The contrasting directions and locations of these threshold-specific associations also show that the profiles cannot be arranged on a single favorable-to-unfavorable axis.

The raising and texture association retained its direction toward poorer grades in the profile-only models of every specification, remaining above 1 at every cutpoint, although its proportional form and precision were not retained when the nonsweetener classes of the marker substances were restored, and its direction was no longer consistent across cutpoints once the restored count was added (Supplementary Table S15). For the oxidation and color-retention profile, the inverse direction was retained, although its cutpoint-specific form was less stable. For the coating and structure profile, the point estimate of the less sparse C–E versus A–B contrast remained above 1 in the primary, gas-collapse, structurally reduced, and nonsweetener-function restoration analyses and in the binary and four-level re-estimations (1.14 to 2.30); however, its magnitude was imprecise and specification-dependent, and its confidence interval included 1 in the gas-collapse and structurally reduced analyses (Table 2; Section 3.4; Supplementary Tables S8–S10 and S15).

### 4.2. Comparison with Other Studies

Previous market studies have identified recurring additive structure in France, Hong Kong, Brazil, and five Latin American countries [1,2,3,4]. Furthermore, functional-class co-occurrence has been documented. Among 1118 products from Brazil targeted at infants and children, functional classes frequently co-occurred; the authors noted that some functional classes exhibit comparable or complementary functions and may therefore be jointly used [5]. A nutritional evaluation of 298 plant-based meat and sausage analogs on the German market computed Nutri-Score grades and found thickeners to be the most frequent additive functional class, present in >60% of products [37]. The present study extends this literature in two ways. Latent profiles are estimated at the product level from Codex-mapped functional-class indicators, whereas the earlier studies grouped the additives or classes themselves; the resulting profiles are carried into a model of a nutrient profile outcome, with the sampling uncertainty of the nonboundary first-step measurement parameters propagated into a second set of standard errors (Section 2.7.4).

The closest analytical precedent is a data-driven clustering of 7284 packaged products in Uruguay, which identified 19 clusters from nutrient composition and additive disclosure and reported that additive disclosure inconsistently aligned with nutrient profiles [9]. The present design separates the two information sources according to construction: the measurement model employed only functional-class indicators, and nutrient information entered only through the distal outcome. The finding that the three principal profile contrasts retained their directions with the count term included and that the three interpreted profiles were associated with various parts of the grade scale supports the conclusion of Ares et al. [9] that additive disclosure inconsistently aligns with nutrient composition. A related concern has been raised from the nutrient side: in the Italian market, nutritional composition and nutrient profile grade broadly varied within the ultra-processed category, leading the authors to argue that health relevance should be assessed by food group and subgroup alongside processing status [11]. Accounting for the food subgroup in the present model supports that emphasis while addressing a different, formulation-based question.

Within Turkey, previous label studies have reported prevalence by additive class or nutritional outcome. Sodium-containing additives were reported in 53.5% of 2975 packaged products [14] and phosphorus-based additives in 58.3% of 3176 analyzed products, with heterogeneous declaration practice [15]. Şimşek et al. [16] reported grades D or E for 61.6% of 1365 ultra-processed products under an earlier Nutri-Score specification, whereas Parlak-Yetişen and Dikmen [17], applying the updated algorithm, indicated grades D or E for 70.0% of 781 child-targeted products. Grades D or E accounted for 77.3% of the present distal sample; however, as the samples and Nutri-Score specifications vary, a direct prevalence comparison is not appropriate. The present distal sample was initially selected for measurement eligibility, which necessitated at least one retained INS-linked functional-class indicator, and subsequently for distal Nutri-Score eligibility. This study adds a pattern-level view: which Codex-mapped functional classes co-occur within products across the sampled inventory and how the resulting profiles relate to the Nutri-Score grade after accounting for food subgroup.

### 4.3. Interpretation

Because the profiles were strongly structured by food subgroup (Cramér’s V, 0.684), these associations should be interpreted as conditional, descriptive formulation-pattern contrasts after accounting for food-subgroup structure and not as unconditional nutritional rankings of the profiles. Because food categories shape both technological requirements and nutrient composition, the associations do not establish formulation effects independent of food-category composition. Within this context, the profiles admit a technological reading, provided as interpretation rather than as an evaluated mechanism: the raising and texture profile integrates raising- and texture-related functions; the coating and structure profile combines coating-, structuring-, and bulk-related functions; and the oxidation and color-retention profile incorporates antioxidant-, sequestrant-, and color-retention functions. Under modal assignment, the composition of the profiles is consistent with this reading: the raising and texture profile drew 50.6% of its products from the confectionery, biscuits and snacks group; the coating and structure profile was most common in dairy products and milk-based beverages (31.5%) and confectionery (19.9%); and the oxidation and color-retention profile was dispersed across confectionery (20.2%), breakfast spreads and preserves (16.0%), preserved foods (13.5%), and bakery, cereal and pasta products (11.5%) (Supplementary Table S12). The reverse reading, in which product categories determine both the functional requirements and the composition, is partly addressed by the subgroup intercept but cannot be excluded from the between-subgroup component of the associations. These findings reveal declared formulation patterns and do not establish causal effects, additive exposure, or health consequences.

### 4.4. Strengths and Limitations

The main strengths of this study were the explicit INS-based mapping procedure, version-controlled Codex classification, audited derivation chain from workbook to indicator matrix, prespecified primary analyses, and explicit assessment of first-step measurement-parameter uncertainty in the distal analysis.

This study had several limitations. First, the profiles relied on label-declared additives that could be resolved to specific INS entries; class-name-only declarations reduced the ascertainment of the full Codex functional profile, and label-based data cannot capture undeclared additive use or additive quantities. However, directly incorporating the explicitly declared class left the primary profile solution essentially unchanged (Section 3.6). Measurement eligibility also selected products with at least one retained exact INS-linked functional-class indicator, which differed from excluded products in food-group composition, ingredient number, and calculated grade (Supplementary Table S12, Panel H), so the associations apply to products meeting the exact INS-linked measurement-eligibility rule rather than to the whole inventory. Label transcription was subject to full second-investigator verification against the package images rather than independent duplicate coding, and no quantitative inter-rater statistic is available for transcription; for the mapping step, however, a revision-stage blind re-mapping audit of the 87 verified codes showed exact agreement (Section 2.8). Second, the Codex-listed purposes were indicative rather than exhaustive, and a single INS entry potentially contributed to multiple functional-class indicators, which induced structural dependence. The gas-related profile was mapping-sensitive and was not substantively interpreted, and residual local dependence more generally remained: 203 out of 325 indicator pairs exceeded the residual threshold, the alternative model freed only the four largest dependencies, the structural reduction removed only the gas-related redundancy, and a measurement model accounting for all residual dependencies was not attempted, because freeing many direct effects would introduce a far more complex and weakly constrained parameterization (Supplementary Tables S7 and S8). The measurement model also used common class priors across food subgroups; subgroup adjustment in the distal model does not test this measurement-stage restriction. The structural model accounted for food subgroup only. Retail channel, manufacturer clustering, and nutrient-composition strata were not modeled; brand-level dependence was assessed only by a cluster bootstrap based on brand names recorded as text (Section 3.8); and beverage status was examined only through the post-primary exclusion of beverage-branch products (Section 3.7). The profile coefficients are therefore descriptive conditional contrasts rather than formulation effects independent of the products in which those formulations occur. In addition, the outcome-motivated substance-level exclusion of sweetener and polyol markers altered the measurement construct itself and is therefore a design limitation rather than only a technical mapping rule; restoring the nonsweetener classes of these substances retained the directions of the three principal associations in the profile-only model but changed the class structure and the form and precision of the raising and texture association (Section 3.6). Codex mapping standardizes the vocabulary used to represent declared functional classes; it does not imply that the resulting co-occurrence profiles are transferable across national food supplies, because authorization lists, declaration practices, reformulation, and category composition differ between markets. Third, statistical fit did not uniquely determine the number and exact form of the profiles, and no numerical class-selection threshold was prespecified: the revision-stage structurally reduced specification preferred a four-profile solution that recovered the interpreted profiles nearly exactly, and some threshold-specific associations were specification-sensitive, so the principal interpretation is restricted to direction-level findings supported under both specifications (Section 3.8). Finally, the Nutri-Score was investigator-computed and did not appear on Turkish labels. Fiber was undeclared for 546 products; the raising and texture estimate was nearly unchanged under subgroup-median imputation but attenuated when the analysis was limited to products with declared fiber, so it is directionally consistent but magnitude-sensitive to the zero-point handling of undeclared fiber. Because non-declaration was strongly patterned by food group, differential outcome misclassification from this label-based rule cannot be excluded. In addition, the sampled cross-sectional retail inventory was not nationally representative, and without barcode-by-date linkage, could not identify every temporal reformulation during the collection period (Section 2.2). Therefore, the findings describe formulation patterns in this sampled inventory rather than in the national packaged food supply.

### 4.5. Implications

Three implications follow, all at the level of exploratory supply-side description and subject to external replication. For surveillance, recording the full declaration and resolving it to specific entries provides information that class-name-only records cannot supply. For monitoring, additive counts can offer a dense summary of formulation differences: in the Polish market, products labeled as containing no added sugar declared a mean of 1.6 additives per product compared with 3.8 in products with added sugar [38]. A profile-based description may further capture differences in which functions co-occur that a count alone would miss; however, pattern and count effects could not be clearly separated in these data. For the labeling debate, formulation patterns and nutrient composition represented associated but distinct dimensions of this sampled inventory, aligning with reports that nutrient-based and processing- or formulation-based descriptions capture different properties of the food supply [8,9], and with Sadler et al. [39], who indicated that most processing-based classification systems do not include a quantitative nutritional assessment. Extending these supply-side profiles toward dietary exposure would warrant linkage to consumption data; an exposure assessment of nine priority additives among 433 adults in Istanbul offers a methodological example from Turkey [40].

A subsequent study could extend the present supply-side analysis using a mixed-methods design. Its quantitative component could examine additive number and type, nutritional quality, food category, price positioning, brand type, and retail channel across a broader sample of the Turkish food market. A complementary qualitative component involving relevant stakeholders, such as food manufacturers, product developers, retailers, and where appropriate, consumers, could investigate the technological, commercial, and reformulation considerations underlying these quantitative patterns. Integration of the two components could clarify whether additive-use patterns and nutritional quality differ across market segments and why such differences arise.

## 5. Conclusions

Label-declared additive use in this sampled Turkish retail inventory showed structured co-occurrence across Codex functional classes. Because the profiles were strongly structured by food subgroup, the profile–grade associations are conditional descriptive contrasts and do not show that functional-class profiles independently determine Nutri-Score grade. Across specifications, the raising and texture profile retained its direction toward poorer Nutri-Score grades, although its magnitude and proportional form were specification-sensitive and its direction was no longer consistent across cutpoints when the restored count was added; the oxidation and color-retention profile retained an inverse association at the grade-E boundary, although its threshold-specific form was not stable; and the coating and structure profile remained displaced from the favorable end, with an imprecise and specification-sensitive magnitude. Within the primary specification, these associations retained their directions after the inclusion of functional-class count; however, the limited overlap in count values prevents a clear separation of pattern and count effects. In a direct benchmark in the primary specification, the profile-only model provided a substantially better descriptive fit than the count-only model, whereas adding the count did not improve the profile-only model; when the nonsweetener classes of the marker substances were restored, the count added information beyond the profiles. Formulation patterns and nutrient composition represented associated but distinct dimensions of this sampled inventory. As the indicators were Codex-mapped rather than verified product functions and residual local dependence among them was extensive, the findings should be interpreted as descriptive specification-dependent associations. The profiles describe declared presence, not additive intake, and no health inference is drawn. The findings support recurrent declaration patterns within this sampled inventory but do not establish a validated five-profile taxonomy for the Turkish food supply. Under an outcome-blind structurally reduced specification, four profiles were preferred and the direction of all three principal associations was retained; the same held in the profile-only model when the nonsweetener classes of the marker substances were restored and six classes were preferred.

## Figures and Tables

**Table 1:** Characteristics of the measurement and distal samples.

Characteristic	Measurement Sample	Distal Sample
Products, *n*	3383	2942
Food groups, *n*	12	11
Food subgroups, *n*	67	56
Subgroup size, median (range)	34 (1–329)	35 (1–324)
Subgroups with <10 products, *n*	16	14
Resolved INS entries per product, median (IQR)	3 (1–5)	3 (2–5)
Resolved INS entries per product, range	1–35	1–35
Functional classes per product, median (IQR)	8 (4–11)	8 (4–11)
Functional classes per product, range	1–23	1–23
Latent profile, *n* (%)		
P1 Sparse	1041 (30.8)	903 (30.7)
P2 Oxidation/color retention	845 (25.0)	742 (25.2)
P3 Raising/texture	803 (23.7)	677 (23.0)
P4 Coating/structure	518 (15.3)	447 (15.2)
P5 Carbonation/pressurization	176 (5.2)	173 (5.9)
Nutri-Score grade, *n* (%)		
Grade A	–	111 (3.8)
Grade B	–	98 (3.3)
Grade C	–	460 (15.6)
Grade D	–	928 (31.5)
Grade E	–	1345 (45.7)

IQR, interquartile range; INS, International Numbering System for food additives. The measurement sample comprises products with at least one retained functional-class indicator derived from an exactly resolved INS entry. The distal sample denotes the subset with an applicable and derivable Nutri-Score grade and determinate FVL content. Profile membership is by modal assignment. The Nutri-Score was computed by the investigators from label-derived nutrition and ingredient information and is not displayed on any package. The grade distribution is conditional on the eligibility criteria and is not a prevalence estimate for the Turkish packaged-food supply.

**Table 2:** Associations between latent profiles and Nutri-Score grade in the distal sample (*n* = 2942 products across 56 food subgroups).

Profile	Cutpoint	Model A OR (95% CI)	Model B OR (95% CI)	Reduced Specification, Model A OR (95% CI)
P2 Oxidation/color retention	B–E vs. A	0.93 (0.58–1.50)	0.81 (0.48–1.38)	0.71 (0.57–0.88)
	C–E vs. A–B	1.10 (0.73–1.64)	0.96 (0.60–1.53)	0.71 (0.57–0.88)
	D–E vs. A–C	0.88 (0.66–1.15)	0.76 (0.53–1.10)	0.71 (0.57–0.88)
	E vs. A–D	0.49 (0.37–0.63)	0.42 (0.29–0.60)	0.71 (0.57–0.88)
P3 Raising/texture	Common OR across all four cutpoints	1.99 (1.51–2.63)	1.60 (1.04–2.46)	1.99 (1.51–2.62)
P4 Coating/structure	B–E vs. A	10.87 (3.41–34.68)	8.44 (2.51–28.37)	6.59 (2.45–17.71)
	C–E vs. A–B	1.95 (1.16–3.30)	1.52 (0.81–2.87)	1.36 (0.85–2.19)
	D–E vs. A–C	1.16 (0.83–1.62)	0.91 (0.56–1.48)	0.96 (0.70–1.32)
	E vs. A–D	0.92 (0.67–1.26)	0.72 (0.45–1.15)	0.99 (0.74–1.33)

CI, confidence interval; OR, odds ratio. Reference profile: P1 Sparse. ORs >1 indicate higher odds of poorer Nutri-Score grades. Model A comprises the profile terms; Model B additionally includes the overall functional-class count as a restricted cubic spline. Under the prespecified partial proportional odds specification, P2, P4, and P5 are freed across cutpoints, whereas P3 is constrained to a common OR because the proportional odds assumption is not rejected. Both models encompass a food-subgroup random intercept and use fixed-parameter two-step latent-class estimation. Under modal assignment, only 4 out of 447 P4 products are grade A. Full coefficients, standard errors, and the variance-method comparison are available from the corresponding author on reasonable request. The Profile 5 coefficients are omitted from this table because the profile is mapping-sensitive and not substantively interpreted; they are reported in Supplementary Table S14. Reduced specification: revision-stage 24-indicator, four-class specification (Section 3.8), Model A, with matched classes; its oxidation and color-retention term is common across cutpoints, and full results are in Supplementary Table S10. The P4 B–E vs. A estimate rests on 4 grade-A products in P4 and is shown for completeness.

## Data Availability

The frozen INS-to-functional-class mapping, selected analysis code, key non-identifying analytical outputs, and the mapping-audit summary are provided in the Supplementary Analysis Package (Supplementary Materials). The product-level label dataset is not publicly archived because it contains product, brand, and retailer identifiers; it is available from the corresponding author upon reasonable request, where permitted.

## Supplementary Materials

The following supporting information can be downloaded at https://www.mdpi.com/article/10.3390/foods15213838/s1. Table S1: Food groups and subgroups; Table S2: Nutri-Score distribution by food group; Table S3: Functional-class indicator prevalence; Table S4: Latent-class enumeration and stability; Table S5: Sensitivity of principal profile associations; Table S6: Observed and model-based grade distributions; Table S7: Indicator redundancy and local dependence; Table S8: Gas-indicator collapse analysis; Table S9: Post-primary robustness analyses; Table S10: Reduced-indicator model results; Table S11: Profile-versus-count model comparison; Table S12: Sample ascertainment, selection, and record quality control; Table S13: First-step variance sensitivity; Table S14: Profile 5 associations; Table S15: Nonsweetener-function restoration analysis; Table S16: Brand- and product-level bootstrap; Figure S1: Associations for all five profiles; Figure S2: Proportional-odds sensitivity distributions; Supplementary Analysis Package (ZIP): Frozen INS mapping, selected analysis code, key outputs, and mapping-audit summary.
