# Supplementary material for: Latent Profiles of Codex-Mapped Additive Functional Classes and Their Association with Nutri-Score in a Sampled Turkish Retail Food Inventory
Source: Foods. 2026 Oct 27;15(21):3838. doi: 10.3390/foods15213838 (PMC13762821; doi:10.3390/foods15213838)

Supplementary Materials

Latent Profiles of Codex-Mapped Additive Functional Classes and Their Association with Nutri-Score in a Sampled Turkish Retail Food Inventory

Supplementary Table S1. Food groups and subgroups represented in the two analytical samples (measurement sample n = 3383; distal sample n = 2942).

Food group	Measurement sample, n	Subgroups represented in the measurement sample	Distal sample, n	Subgroups not represented in the distal sample
Processed meat and packaged poultry products	140	• Processed meat products (*sucuk*, salami, sausages, *pastırma*, smoked and cooked meats)<br>• Packaged poultry and other white-meat products	137	–
Dairy products and milk-based beverages	385	• Milk desserts and puddings<br>• Imported, regional and specialty cheeses<br>• Cream cheese and other spreadable cheeses<br>• White brined, fresh and similar cheeses<br>• Flavored and fruit milk drinks<br>• Yoghurt and fermented milk products<br>• Cream, *kaymak* and other milk-fat products<br>• *Kaşar*, sandwich and processed cheeses<br>• Kefir and probiotic milk drinks<br>• Dessert sauces and toppings	301	• White brined, fresh and similar cheeses<br>• *Kaşar*, sandwich and processed cheeses
Bakery, cereal and pasta products	449	• Baking ingredients and aids<br>• Packaged bread and bakery products<br>• Breakfast cereals<br>• Pasta, noodles, *mantı* and other dry dough products<br>• Oats, muesli, granola and cereal mixes	442	–
Non-alcoholic beverages	389	• Iced tea, iced coffee and other still beverages<br>• Juices, nectars, lemonade, *şalgam*, *boza* and other still drinks<br>• Flavored natural mineral water<br>• *Gazoz* and other flavored carbonated drinks<br>• Energy drinks<br>• Coffee and coffee-based drinks<br>• Coffee whiteners, *salep* and instant powdered drinks<br>• Drinking water, spring water and natural mineral water<br>• Cola drinks<br>• Tonic water<br>• Non-alcoholic malt beverages<br>• Tea products (loose-leaf, bagged and herbal)	328	• Drinking water, spring water and natural mineral water<br>• Tea products (loose-leaf, bagged and herbal)
Breakfast spreads, preserves and related products	283	• Table olives and olive-based products<br>• Jams, marmalades and fruit-based spreads<br>• Chocolate spreads and nut pastes<br>• Breakfast sauces and savory spreads<br>• Tahini, *pekmez*, halva and related products	282	–

Food group	Measurement sample, n	Subgroups represented in the measurement sample	Distal sample, n	Subgroups not represented in the distal sample
Confectionery, biscuits and snack foods	932	• Chocolate, wafers and confectionery bars<br>• Biscuits, crackers and cakes<br>• Crisps and extruded savory snacks<br>• Sugar confectionery and chewing gum<br>• Dried fruit<br>• Nuts and seeds<br>• Roasted pulse and cereal snacks	879	–
Special-diet, plant-based and special-purpose products	230	• Plant-based alternatives<br>• Sugar-free and no-added-sugar products<br>• Gluten-free products<br>• Sports foods and food supplements<br>• Plant protein ingredients	124	• Sports foods and food supplements<br>• Plant protein ingredients
Infant formula and complementary foods	63	• Complementary foods for infants and young children<br>• Infant formula and follow-on formula	Not represented	• Complementary foods for infants and young children<br>• Infant formula and follow-on formula
Oils, margarines and fat spreads	37	• Margarines, fat spreads and vegetable or specialty oils	31	–
Preserved foods and ready meals	157	• Pickled vegetables<br>• Canned vegetables and pulses<br>• Canned and ready-to-serve soups<br>• Tomato products and pastes<br>• Canned fish and seafood	149	–
Sauces, condiments and seasonings	213	• Table sauces, seasonings, condiments and soup mixes<br>• Cooking sauces<br>• Salad dressings and vinegar-based condiments	194	–
Frozen foods	105	• Frozen desserts, baklava, *künefe* and related products<br>• Frozen fish and seafood<br>• Frozen pizzas<br>• Frozen filled savory pastries<br>• Frozen dough<br>• Frozen bakery products<br>• Frozen flatbreads<br>• Frozen puff pastry<br>• Frozen fruit<br>• Frozen potato products	75	• Frozen pizzas<br>• Frozen bakery products<br>• Frozen flatbreads
All food groups	3383	67 subgroups	2942	11 subgroups not represented; 56 subgroups represented

Retained subgroups may contain fewer products after product-level distal eligibility. Labels are English equivalents of the source Turkish categories; Turkish product names remain italicized where no exact English equivalent is available. Eligibility criteria are given in Section 2.6 and Fig. 1.

Supplementary Table S2. Nutri-Score grade distribution by food group in the distal sample (n = 2942).

Food group	*n*	Grade A, n (%)	Grade B, n (%)	Grade C, n (%)	Grade D, n (%)	Grade E, n (%)
Processed meat and packaged poultry products	137	5 (3.6)	7 (5.1)	23 (16.8)	62 (45.3)	40 (29.2)
Dairy products and milk-based beverages	301	2 (0.7)	24 (8.0)	88 (29.2)	157 (52.2)	30 (10.0)
Bakery, cereal and pasta products	442	57 (12.9)	15 (3.4)	114 (25.8)	115 (26.0)	141 (31.9)
Non-alcoholic beverages	328	0 (0.0)	20 (6.1)	61 (18.6)	101 (30.8)	146 (44.5)
Breakfast spreads, preserves and related products	282	2 (0.7)	0 (0.0)	21 (7.4)	130 (46.1)	129 (45.7)
Confectionery, biscuits and snack foods	879	7 (0.8)	2 (0.2)	32 (3.6)	167 (19.0)	671 (76.3)
Special-diet, plant-based and special-purpose products	124	4 (3.2)	8 (6.5)	53 (42.7)	32 (25.8)	27 (21.8)
Oils, margarines and fat spreads	31	0 (0.0)	0 (0.0)	9 (29.0)	14 (45.2)	8 (25.8)
Preserved foods and ready meals	149	17 (11.4)	12 (8.1)	33 (22.1)	54 (36.2)	33 (22.1)
Sauces, condiments and seasonings	194	15 (7.7)	3 (1.5)	16 (8.2)	73 (37.6)	87 (44.8)
Frozen foods	75	2 (2.7)	7 (9.3)	10 (13.3)	23 (30.7)	33 (44.0)
All food groups	2942	111 (3.8)	98 (3.3)	460 (15.6)	928 (31.5)	1345 (45.7)

Values are counts with row percentages. Nutri-Score was computed by the investigators from label-derived nutrition and ingredient information using the updated 2023 algorithm. Percentages may not sum to exactly 100 because of rounding.

Supplementary Table S3. Prevalence of the 26 Codex-derived functional-class indicators in the measurement sample (n = 3383).

Codex-derived functional class	Products, n	Products, %
Acidity regulator	2293	67.8
Emulsifier	2218	65.6
Stabilizer	2166	64.0
Sequestrant	2033	60.1
Thickener	1959	57.9
Antioxidant	1718	50.8
Color-retention agent	1391	41.1
Preservative	1201	35.5
Humectant	1053	31.1
Glazing agent	1026	30.3
Color	996	29.4
Anticaking agent	974	28.8
Emulsifying salt	967	28.6
Raising agent	916	27.1
Flour-treatment agent	812	24.0
Bulking agent	623	18.4
Gelling agent	601	17.8
Carrier	596	17.6
Firming agent	575	17.0
Foaming agent	554	16.4
Antifoaming agent	440	13.0
Flavor enhancer	249	7.4
Bleaching agent	185	5.5
Propellant	177	5.2
Packaging gas	177	5.2
Carbonating agent	176	5.2

An indicator takes the value one when at least one exactly resolved INS entry retained after the prespecified substance-level marker exclusion carries that functional class in the frozen mapping. Sweetener is not represented because marker entries were excluded before functional-class mapping. Counts are products rather than declarations, and a product may contribute to several functional-class indicators.

Supplementary Table S4. Latent class enumeration and stability diagnostics for one to twelve classes (n = 3383 products, 26 binary indicators).

Classes	Log-likelihood	Parameters	AIC	BIC	aBIC	Entropy	Smallest class, n (%)	Boundary conditional probabilities, n	Half-sample matches (of 50 splits)	Mean |difference|, half-sample
1	-44,866.7	26	89,785.5	89,944.8	89,862.1	1.000	3383 (100.0)	0	–	–
2	-38,608.6	53	77,323.1	77,647.8	77,479.4	0.911	1597 (47.2)	1	–	–
3	-35,504.0	80	71,167.9	71,658.1	71,403.9	0.955	895 (26.5)	3	50/50	0.0142
4	-33,637.3	107	67,488.7	68,144.2	67,804.2	0.967	699 (20.7)	9	47/50	0.0238
5	-32,110.7	134	64,489.3	65,310.3	64,884.5	0.980	176 (5.2)	21	42/50	0.0315
6	-30,931.6	161	62,185.2	63,171.6	62,660.0	0.978	176 (5.2)	34	27/50	0.0245
7	-29,849.1	188	60,074.2	61,226.0	60,628.6	0.986	176 (5.2)	52	33/50	0.0257
8	-29,145.5	215	58,721.1	60,038.3	59,355.1	0.980	176 (5.2)	60	29/50	0.0371
9	-28,612.6	242	57,709.1	59,191.8	58,422.8	0.986	176 (5.2)	71	–	–
10	-28,175.9	269	56,889.7	58,537.7	57,683.0	0.981	176 (5.2)	79	–	–
11	-27,711.8	296	56,015.5	57,829.0	56,888.5	0.985	172 (5.1)	89	–	–
12	-27,268.7	323	55,183.3	57,162.2	56,135.9	0.986	60 (1.8)	106	–	–

Supplementary Table S4 (continued). Enumeration and stability diagnostics for the revision-stage reduced 24-indicator specification (Section 2.7.5); primary 26-indicator results are shown above.

Classes	Log-likelihood	Parameters	BIC	Entropy	Smallest class %	Boundary parameters	Half-sample, primary criterion (Variant B)	Half-sample, half-to-half
1	-43,480.6	24	87,156.3	1.000	100.0	0		
2	-37,232.3	49	74,862.7	0.907	47.7	1		
3	-34,334.7	74	69,270.8	0.964	25.1	2	50/50	50/50
4	-32,524.5	99	65,853.5	0.976	16.7	5	50/50	50/50
5	-31,346.6	124	63,700.8	0.975	15.5	15	6/50	8/50
6	-30,171.3	149	61,553.5	0.983	11.4	29	37/50	27/50
7	-29,472.4	174	60,358.7	0.978	8.7	37	21/50	22/50
8	-28,907.7	199	59,432.6	0.979	5.6	40	9/50	12/50
9	-28,408.5	224	58,637.4	0.980	5.4	48		
10	-27,924.9	249	57,873.3	0.982	4.4	54		
11	-27,557.0	274	57,340.8	0.985	3.3	70		
12	-27,191.3	299	56,812.4	0.977	4.2	78		

AIC, Akaike information criterion; BIC, Bayesian information criterion; aBIC, sample-size-adjusted BIC. Half-sample diagnostics were evaluated for three to eight classes; dashes indicate not evaluated. Variant B matched each half-sample to the full-sample solution by mutual nearest neighbors on mean absolute conditional-probability differences (400 full-sample and 120 half-sample starts); a split counted as matched only when every class was recovered in both halves. Half-to-half matching used 60 starts per half; in the primary model, half-to-half recoveries for three to eight classes were 50/47/40/26/24/33 of 50. Boundary counts are class-specific indicator probabilities estimated at zero or one; mean absolute differences summarize matched probability vectors. Bootstrap likelihood-ratio and replication diagnostics are available on reasonable request.

Supplementary Table S5. Selected sensitivity estimates for the three principal profile–Nutri-Score associations, expressed as odds ratios with 95% confidence intervals (distal sample n = 2942 unless stated in the column heading).

Profile	Cutpoint	Primary	Subgroup fixed effects	Alternative measurement model	Four profiles (26 indicators)	Six profiles (26 indicators)	Polyols re-included	CNO products excluded (n = 2269)
P3 Raising / texture	common across cutpoints	1.99 (1.51 to 2.63)	1.99 (1.50 to 2.63)	1.75 (1.33 to 2.30)	1.90 (1.44 to 2.51)	1.65 (1.19 to 2.28)	1.69 (1.27 to 2.23)	1.84 (1.29 to 2.62)
P2 Oxidation / color retention	E vs A–D	0.49 (0.37 to 0.63)	0.48 (0.37 to 0.63)	0.49 (0.37 to 0.65)	0.71 (0.56 to 0.89)	0.40 (0.30 to 0.54)	0.50 (0.38 to 0.66)	0.49 (0.36 to 0.67)
P4 Coating / structure	B–E vs A	10.87 (3.41 to 34.68)	11.06 (3.44 to 35.50)	22.49 (5.23 to 96.76)	8.63 (3.13 to 23.80)	9.77 (2.65 to 36.00)	11.12 (3.61 to 34.31)	7.45 (1.89 to 29.32)

ORs are relative to P1 Sparse; values above one indicate poorer grades. Subgroup fixed effects replace the random intercept. The alternative measurement model frees four indicator-pair direct effects and uses conditional standard errors. The four- and six-profile analyses use the original 26 indicators, with profiles matched by Hungarian assignment. Polyol re-inclusion used a locked warm start because unrestricted estimation did not meet comparability criteria. The class-name-only (CNO) exclusion had a non-positive-definite Hessian; its intervals are descriptive only. The sparse P4 B–E versus A estimate (4 grade-A products in P4) is shown for completeness; the less sparse C–E versus A–B contrast (1.95, 1.16 to 3.30, in the primary model) is reported across specifications in Supplementary Tables S8 to S10 and S15.

Supplementary Table S6. Observed, model-standardized marginal and zero-random-effect conditional Nutri-Score grade distributions by latent profile (n = 2942). Panel B is derived from the adopted partial proportional odds model reported in Table 2 of the main text and corresponds to Figure 4, panel b; Panels C and D are derived from the proportional odds form of Model A (Section 2.7.3) and are reported as a sensitivity version (Supplementary Figure S2).

Panel A. *Observed distribution under modal profile assignment, n (row %).*

Profile	*n*	Grade A	Grade B	Grade C	Grade D	Grade E
P1	903	50 (5.5)	29 (3.2)	125 (13.8)	270 (29.9)	429 (47.5)
P2	742	46 (6.2)	21 (2.8)	137 (18.5)	323 (43.5)	215 (29.0)
P3	677	10 (1.5)	7 (1.0)	64 (9.5)	133 (19.6)	463 (68.4)
P4	447	4 (0.9)	25 (5.6)	97 (21.7)	161 (36.0)	160 (35.8)
P5	173	1 (0.6)	16 (9.2)	37 (21.4)	41 (23.7)	78 (45.1)

Panel B. *Model-standardized marginal probabilities (%) from the adopted partial proportional odds model, with 95% confidence intervals.*

Profile	Grade A	Grade B	Grade C	Grade D	Grade E
P1	10.3 (6.4 to 15.6)	5.4 (3.6 to 7.9)	22.1 (18.1 to 26.3)	31.6 (27.5 to 35.5)	30.7 (23.5 to 38.2)
P2	10.8 (6.6 to 16.5)	4.0 (2.2 to 6.2)	25.1 (20.4 to 30.0)	39.0 (33.4 to 44.0)	21.1 (15.0 to 27.9)
P3	6.2 (3.5 to 10.4)	3.7 (2.3 to 5.8)	17.6 (13.7 to 21.9)	31.1 (27.0 to 34.9)	41.4 (32.6 to 49.8)
P4	1.5 (0.5 to 4.7)	8.6 (4.7 to 13.6)	25.3 (19.7 to 31.2)	35.1 (29.5 to 40.5)	29.5 (21.9 to 37.4)
P5	1.2 (0.1 to 7.8)	13.2 (4.9 to 25.3)	19.4 (11.3 to 28.2)	17.4 (11.6 to 22.0)	48.8 (30.5 to 67.5)

Panel C. *Model-standardized marginal probabilities (%) from the proportional odds form (sensitivity), with 95% confidence intervals.*

Profile	Grade A	Grade B	Grade C	Grade D	Grade E
P1	7.8 (4.8 to 12.4)	5.9 (4.1 to 8.1)	23.2 (19.1 to 27.0)	33.5 (29.5 to 37.0)	29.5 (22.6 to 37.3)
P2	10.4 (6.5 to 15.9)	7.2 (5.2 to 9.5)	25.7 (21.8 to 29.6)	32.5 (28.4 to 36.2)	24.1 (17.7 to 31.3)
P3	4.5 (2.5 to 7.7)	3.9 (2.5 to 5.7)	17.9 (13.7 to 22.0)	33.0 (29.0 to 36.3)	40.8 (32.3 to 49.7)
P4	7.2 (4.2 to 11.8)	5.6 (3.8 to 7.7)	22.4 (18.2 to 26.4)	33.6 (29.7 to 37.0)	31.2 (23.7 to 39.5)
P5	4.4 (1.6 to 10.3)	3.8 (1.7 to 7.2)	17.7 (10.3 to 25.7)	32.9 (26.6 to 36.4)	41.1 (24.7 to 59.0)

Panel D. *Conditional probabilities at a zero random effect (%) from the proportional odds form (sensitivity), with 95% confidence intervals.*

Profile	Grade A	Grade B	Grade C	Grade D	Grade E
P1	3.1 (1.8 to 5.2)	3.6 (2.2 to 5.9)	24.4 (17.0 to 32.6)	47.3 (42.5 to 49.7)	21.6 (14.1 to 31.3)
P2	4.5 (2.7 to 7.6)	5.1 (3.2 to 8.2)	30.5 (22.7 to 38.4)	44.2 (36.8 to 48.6)	15.6 (9.8 to 23.4)
P3	1.5 (0.9 to 2.6)	1.8 (1.0 to 3.1)	14.5 (9.3 to 21.7)	45.8 (39.0 to 49.2)	36.4 (25.1 to 49.6)
P4	2.8 (1.6 to 4.7)	3.3 (1.9 to 5.4)	22.7 (15.4 to 31.1)	47.7 (43.3 to 49.8)	23.6 (15.2 to 34.5)
P5	1.5 (0.5 to 4.4)	1.8 (0.6 to 5.0)	14.3 (5.6 to 29.5)	45.6 (29.9 to 49.3)	36.9 (16.5 to 63.4)

P1, Sparse; P2, Oxidation/color retention; P3, Raising/texture; P4, Coating/structure; P5, Carbonation/pressurization. Panel B intervals are based on 4000 multivariate-normal draws of the first-step-corrected covariance (conditional covariance plus the covariance of 100 first-step refits); one draw in which a profile's effective cutpoint sequence was not strictly increasing was excluded. Panels C and D intervals are based on 4000 parametric draws with the percentile method (Section 2.7.3).

Supplementary Table S7. Structural redundancy and selected class-conditional local-dependence diagnostics among the 26 Codex-derived functional-class indicators in the measurement sample (*n* = 3383).

Panel A. *Indicator pairs with strong marginal overlap (phi at least 0.60).*

Indicator pair	n (both)	n (discordant)	Phi	Jaccard
Propellant / Packaging gas	177	0	1.000	1.000
Propellant / Carbonating agent	176	1	0.997	0.994
Packaging gas / Carbonating agent	176	1	0.997	0.994
Stabilizer / Thickener	1928	269	0.841	0.878
Antioxidant / Color-retention agent	1366	377	0.793	0.784
Bulking agent / Carrier	498	223	0.777	0.691
Anticaking agent / Raising agent	785	320	0.766	0.710
Acidity regulator / Sequestrant	1966	394	0.760	0.833
Humectant / Emulsifying salt	802	416	0.708	0.658
Glazing agent / Bulking agent	612	425	0.702	0.590
Sequestrant / Antioxidant	1589	573	0.672	0.735
Sequestrant / Color-retention agent	1361	702	0.644	0.660
Glazing agent / Gelling agent	562	503	0.639	0.528

Panel B. *Deterministic implications, in which the consequent indicator is present in every product carrying the antecedent.*

Indicator (antecedent)	*n*	Always co-occurring indicators
Emulsifying salt	967	Acidity regulator
Raising agent	916	Acidity regulator
Gelling agent	601	Stabilizer; Thickener
Bleaching agent	185	Antioxidant; Preservative; Flour-treatment agent
Propellant	177	Preservative; Foaming agent; Packaging gas
Packaging gas	177	Preservative; Foaming agent; Propellant
Carbonating agent	176	Preservative; Foaming agent; Propellant; Packaging gas

Panel C. *Selected class-conditional local-dependence diagnostics from the primary five-class model: the five largest bivariate residuals of the 325 indicator pairs, and the three gas-related pairs.*

Indicator pair	Rank of 325	Bivariate residual	Bootstrap 95th centile
Stabilizer / Thickener	1	745.8	1.79
Flour-treatment agent / Bleaching agent	2	552.2	3.47
Antioxidant / Color-retention agent	3	475.2	1.73
Glazing agent / Antifoaming agent	4	421.3	1.34
Preservative / Bleaching agent	5	366.9	3.22
Propellant / Packaging gas	183	2.0	0.00
Propellant / Carbonating agent	324	0.0	0.00
Packaging gas / Carbonating agent	325	0.0	0.00

Supplementary Table S7 (continued). Observed deterministic indicator implications (17 ordered pairs; revision-stage structural-reduction rule, Section 2.7.5). Fifteen of the 17 implications were forced by the frozen mapping at the code level; two were empirical product-level implications. For each ordered pair, the table reports whether the frozen Codex mapping forces the child indicator to imply the parent at the code level, and the product counts. Only the propellant, packaging-gas, and carbonating-agent trio additionally satisfied the product-level near-equivalence criterion and was merged; all other indicators below were retained separately.

Child indicator	Parent indicator	Products, child	Products, parent	INS codes, child	Code-level forced
emulsifying_salt	acidity_regulator	967	2293	21	True
raising_agent	acidity_regulator	916	2293	16	True
gelling_agent	stabilizer	601	2166	10	True
gelling_agent	thickener	601	1959	10	True
bleaching_agent	antioxidant	185	1718	4	True
bleaching_agent	preservative	185	1201	4	True
bleaching_agent	flour_treatment_agent	185	812	4	True
propellant	foaming_agent	177	554	2	True
propellant	packaging_gas	177	177	2	True
packaging_gas	foaming_agent	177	554	2	True
packaging_gas	propellant	177	177	2	True
carbonating_agent	preservative	176	1201	1	True
carbonating_agent	foaming_agent	176	554	1	True
carbonating_agent	propellant	176	177	1	True
carbonating_agent	packaging_gas	176	177	1	True
propellant	preservative	177	1201	2	False
packaging_gas	preservative	177	1201	2	False

Phi and Jaccard measure marginal overlap, not conditional local independence; bivariate residuals assess within-class dependence against bootstrap 95th-centile thresholds. The four largest residuals were freed in the alternative measurement model; gas-related pairs had near-complete marginal overlap but negligible residuals. A deterministic implication was mapping-forced when every INS entry carrying the antecedent also carried the consequent; the propellant-preservative exception arose because the single product declaring INS 941 without INS 290 declared a separate preservative.

Supplementary Table S8. Sensitivity of the latent profile solution and profile–Nutri-Score associations to collapsing three near-redundant gas-related indicators into a single composite indicator, with the number of classes fixed at five (the reduced specification with re-selected class number is reported in Supplementary Table S10).

Panel A. *Profile recovery and distal associations.*

Result	Primary 26-indicator model	Gas-collapsed 24-indicator model
Indicators in the measurement model	26	24
Profile 1 (sparse) matched conditional-probability correlation	–	0.750 (the sparse class divides into two)
Profile 2 matched conditional-probability correlation	–	0.995
Profile 3 matched conditional-probability correlation	–	0.986
Profile 4 matched conditional-probability correlation	–	0.994
Profile 5 matched conditional-probability correlation	–	0.374 (not recovered)
Profile 2, proportional odds test, Holm-adjusted p	<0.001 (freed across cutpoints)	0.10 (proportional retained)
Profile 3, proportional odds test, Holm-adjusted p	0.060 (proportional retained)	0.24 (proportional retained)
Profile 4, proportional odds test, Holm-adjusted p	<0.001 (freed across cutpoints)	<0.001 (freed across cutpoints)
Profile 2, oxidation and color retention	0.49 (0.37 to 0.63) at the E versus A to D cutpoint	0.76 (0.59 to 0.98), common across cutpoints
Profile 3, raising and texture	1.99 (1.51 to 2.63), common across cutpoints	2.27 (1.66 to 3.10), common across cutpoints
Profile 4, coating and structure, B to E versus A	10.87 (3.41 to 34.68)	5.37 (1.87 to 15.45)
Profile 4, coating and structure, remaining three cutpoints (C–E vs A–B; D–E vs A–C; E vs A–D)	1.95 (1.16 to 3.30); 1.16 (0.83 to 1.62); 0.92 (0.67 to 1.26)	1.14 (0.69 to 1.90); 0.95 (0.68 to 1.35); 1.12 (0.80 to 1.55)
Food-subgroup standard deviation	1.694	1.642
Intraclass correlation	0.466	0.450
Classification entropy	0.980	0.975

Panel B. *Smallest-class trajectory across the class enumeration.*

Classes	Primary 26-indicator model, smallest class n (%)	Gas-collapsed 24-indicator model, smallest class n (%)
3	895 (26.5)	849 (25.1)
4	699 (20.7)	565 (16.7)
5	176 (5.2)	524 (15.5)
6	176 (5.2)	384 (11.4)
7	176 (5.2)	294 (8.7)
8	176 (5.2)	191 (5.6)
9	176 (5.2)	184 (5.4)
10	176 (5.2)	148 (4.4)
11	172 (5.1)	113 (3.3)
12	60 (1.8)	143 (4.2)

Pearson correlations were calculated across 23 shared indicators. Hungarian matches were P1–N4 (0.750), P2–N1 (0.995), P3–N3 (0.986), P4–N5 (0.994), and P5–N2 (0.374); r(P1, N2) = 0.742 further illustrates reference-profile splitting.

This protocol-amendment analysis, specified after completion of the primary analyses, collapsed three gas indicators while retaining five classes and using Model A; it does not replace the primary solution. The same 176 INS-290 products formed the smallest class in the primary five- to ten-class solutions but not after collapse. BIC declined throughout enumeration in both specifications, so Panel B is descriptive.

Supplementary Table S9. Additional post-primary robustness analyses of the three principal profile–Nutri-Score associations (odds ratios with 95% confidence intervals).

Panel A. *Principal estimates.*

Analysis	Sample / outcome	Measurement comparison	P3 (common term unless stated)	P2 (E vs A–D unless stated)	P4 (B–E vs A unless stated)
Declared-class incorporation	*n* = 3383 measurement; *n* = 2942 distal; five-level PPO	agreement 1.000; mean |Δρ| ≤ 0.0011	1.99 (1.51 to 2.63)	0.49 (0.37 to 0.63)	10.93 (3.40 to 35.16)
Nonsweetener marker-class restoration (fixed five-class solution)	*n* = 3383 measurement; *n* = 2942 distal; five-level PPO	agreement 0.954; mean |Δρ| ≤ 0.0418	1.69 (1.27 to 2.24)	0.50 (0.38 to 0.66)	11.13 (3.61 to 34.32)
Beverage-branch products excluded	*n* = 2549 distal; five-level PPO (PO re-tested)	measurement model fixed	four cutpoints: 2.25 / 2.62 / 1.59 / 2.08	0.49 (0.37 to 0.65)	9.50 (2.93 to 30.78)
Favorable-grade collapse (binary A–B vs C–E)	*n* = 2942 distal; binary two-step mixed model	measurement model fixed	2.91 (1.51 to 5.59) (C–E vs A–B)	0.98 (0.63 to 1.54) (C–E vs A–B)	1.86 (1.05 to 3.32) (C–E vs A–B)

Panel B. *Measurement diagnostics for the analyses that re-estimated the measurement model.*

Analysis	Modification scope	Products with a changed indicator vector	Indicator cells changed	Matched modal agreement	Mean / max |Δρ| (largest class)
Declared-class incorporation	1623 inventory / 1591 measurement class-name-only records	33	33 of 87,958 (0.038%)	1.000	0.0011 / 0.0157
Nonsweetener marker-class restoration (fixed five-class solution)	19 marker code stems; 387 products carrying markers	320	617 of 87,958 (0.701%)	0.954	0.0418 / 0.4489

These analyses were post-primary and not preregistered; confidence intervals use conditional observed-information variances. Declared-class incorporation added explicitly named functions without inferring unresolved INS identities. Marker-function restoration retained the original measurement sample and five-class solution; full re-enumeration is reported in Supplementary Table S15. Both measurement re-estimations used locked warm starts, and the 26 marker-only products were not re-admitted. Beverage-branch exclusion removed 393 products and yielded nonproportional P3 estimates, all above one. The binary model coded C–E as 1 and A–B as 0, so all three entries in that row are the C–E versus A–B contrast (primary partial proportional odds P4: 1.95, 1.16 to 3.30; four-level ordinal: 2.30, 1.33 to 3.99). ρ denotes the class-conditional indicator probabilities, and |Δρ| their absolute change relative to the primary model.

Supplementary Table S10. Revision-stage 24-indicator, four-class analysis (*n* = 3383 measurement; *n* = 2942 distal), specified before execution but not prespecified. Panel A summarizes class selection (full record in Supplementary Table S4, continued); Panels B to D report profile matching, distal coefficients, and cutpoint-specific comparisons; Panel E presents leave-one-food-group-out estimates for the P2 grade-E contrast. The principal interpreted contrasts were P2 at grade E, P3 common, and P4 at C–E versus A–B; other cutpoints may differ in direction. Non-estimable fits (non-convergence, non-positive-definite Hessian, or non-increasing cutpoints) are labeled and not replaced. Panel E was not repeated for the reduced model because P2 was proportional.

Panel A. *Class selection (24 indicators): three and four classes were fully recovered in both half-sample variants (50/50 and 50/50), and only the four-class solution recovered the interpreted primary profiles; five classes were recovered in 6/50 (Variant B) and 8/50 (half-to-half) splits. Four classes were selected. The full one-to-twelve-class record is given in Supplementary Table S4 (continued).*

Panel B. *Matching of the reduced four-class solution to the primary profiles (Pearson correlation over 23 shared indicators; MAD, mean absolute difference).*

Primary profile	Matched correlation	MAD	Modal agreement
P1 Sparse	1.000	0.0004	1041 of 1041
P2 Oxidation/color retention	0.995	0.018	842 of 845
P3 Raising/texture	1.000	0.0010	803 of 803
P4 Coating/structure	0.995	0.026	517 of 518
P5 Carbonation/pressurization	no separate class	–	123 to P2-like, 48 to P4-like, 5 elsewhere (of 176)

Panel C. *Reduced-specification partial proportional odds coefficients (reference: class matched to P1). Only the P4-matched term was freed, and adding the original 26-indicator count did not improve fit (chi-square 3.02, df = 2, p = 0.22).*

Class (matched)	Cutpoint	Proportional	OR (95% CI)
P2-matched	B–E vs. A	yes	0.71 (0.57–0.88)
P2-matched	C–E vs. A–B	yes	0.71 (0.57–0.88)
P2-matched	D–E vs. A–C	yes	0.71 (0.57–0.88)
P2-matched	E vs. A–D	yes	0.71 (0.57–0.88)
P3-matched	B–E vs. A	yes	1.99 (1.51–2.62)
P3-matched	C–E vs. A–B	yes	1.99 (1.51–2.62)
P3-matched	D–E vs. A–C	yes	1.99 (1.51–2.62)
P3-matched	E vs. A–D	yes	1.99 (1.51–2.62)
P4-matched	B–E vs. A	no	6.59 (2.45–17.71)
P4-matched	C–E vs. A–B	no	1.36 (0.85–2.19)
P4-matched	D–E vs. A–C	no	0.96 (0.70–1.32)
P4-matched	E vs. A–D	no	0.99 (0.74–1.33)

Panel D. *Cross-specification comparison of matched-profile contrasts (P5 has no reduced-specification counterpart; its primary estimates are in Supplementary Table S14).*

Primary profile	Cutpoint	Primary OR (95% CI)	Primary proportional	Reduced OR (95% CI)	Reduced proportional	Direction concordant	|Δ log-OR| (same form)
P2	B–E vs. A	0.93 (0.58–1.50)	no	0.71 (0.57–0.88)	yes	yes	
P2	C–E vs. A–B	1.10 (0.73–1.64)	no	0.71 (0.57–0.88)	yes	no	
P2	D–E vs. A–C	0.88 (0.66–1.15)	no	0.71 (0.57–0.88)	yes	yes	
P2	E vs. A–D	0.49 (0.37–0.63)	no	0.71 (0.57–0.88)	yes	yes	
P3	B–E vs. A	1.99 (1.51–2.63)	yes	1.99 (1.51–2.62)	yes	yes	0.001
P3	C–E vs. A–B	1.99 (1.51–2.63)	yes	1.99 (1.51–2.62)	yes	yes	0.001
P3	D–E vs. A–C	1.99 (1.51–2.63)	yes	1.99 (1.51–2.62)	yes	yes	0.001
P3	E vs. A–D	1.99 (1.51–2.63)	yes	1.99 (1.51–2.62)	yes	yes	0.001
P4	B–E vs. A	10.87 (3.41–34.68)	no	6.59 (2.45–17.71)	no	yes	0.501
P4	C–E vs. A–B	1.95 (1.16–3.30)	no	1.36 (0.85–2.19)	no	yes	0.358
P4	D–E vs. A–C	1.16 (0.83–1.62)	no	0.96 (0.70–1.32)	no	no	0.186
P4	E vs. A–D	0.92 (0.67–1.26)	no	0.99 (0.74–1.33)	no	yes	0.078

Panel E. *Leave-one-broad-food-group-out estimates for the oxidation and color-retention grade-E boundary contrast (primary partial proportional odds model; full-sample estimate 0.49, 95% CI 0.37–0.63).*

Excluded food group	n remaining	OR (95% CI), E vs. A–D
Processed meat and packaged poultry products	2805	0.49 (0.38–0.65)
Dairy products and milk-based beverages	2641	0.46 (0.35–0.60)
Bakery, cereal and pasta products	2500	0.51 (0.38–0.68)
Non-alcoholic beverages	2614	non-estimable (point estimate 0.47; Hessian not positive definite; cutpoint sequence not increasing)
Breakfast spreads, preserves and related products	2660	0.53 (0.39–0.71)
Confectionery, biscuits and snack foods	2063	0.48 (0.36–0.66)
Special-diet, plant-based and special-purpose products	2818	0.53 (0.40–0.69)
Oils, margarines and fat spreads	2911	0.48 (0.36–0.62)
Preserved foods and ready meals	2793	0.49 (0.37–0.65)
Sauces, condiments and seasonings	2748	0.46 (0.34–0.61)
Frozen foods	2867	0.46 (0.35–0.60)

Supplementary Table S11. Count-benchmark ladder on the distal sample (n = 2942; food-subgroup random intercept in all models; count = row sum of the 26 primary indicators as a restricted cubic spline; the class-prior parameters are estimated in all four models). Nested likelihood ratio tests: count-only vs. subgroup-only, chi-square 9.36, df = 2, p = 0.009; profile-plus-count vs. count-only, 153.7, df = 13, $p < 0.001$; profile-plus-count vs. profile-only, 1.92, df = 2, p = 0.38. Count-only and profile-only are not nested; BIC prefers profile-only by 64.0.

Model	Log-likelihood	Parameters	AIC	BIC
subgroup-only	-30,621.54	9	61,261.1	61,315.0
count-only	-30,616.86	11	61,255.7	61,321.6
profile-only (Model A)	-30,540.95	22	61,125.9	61,257.6
profile+count (Model B)	-30,539.99	24	61,128.0	61,271.7

Subgroup-specification sensitivity: maximum absolute change in the profile coefficients when food subgroup is represented by 56 fixed effects instead of a random intercept (adopted partial proportional odds specification, log-odds scale).

Profile	Maximum |Δ log-odds|, fixed effects versus random intercept
P2 Oxidation/color retention	0.0077
P3 Raising/texture	0.0018
P4 Coating/structure	0.0166

The fixed-effects specification is the prespecified sensitivity analysis of Section 2.7.5; its principal estimates are given in Supplementary Table S5. P5 estimates are specification-sensitive and are not used for substantive interpretation (Supplementary Table S14).

Supplementary Table S12. Sample ascertainment, selection, and record-level quality control (inventory *n* = 6515). CNO, class-name-only declaration; MNS, modified-starch-only unresolved declaration; FVL, fruit, vegetable, and legume content.

Panel A. *Measurement-sample eligibility by food group (inventory, n = 6515).*

Food group	Ineligible, n	Eligible (measurement), n	Eligible, %
Processed meat and packaged poultry products	60	140	70.0
Dairy products and milk-based beverages	344	385	52.8
Bakery, cereal and pasta products	783	449	36.4
Non-alcoholic beverages	331	389	54.0
Breakfast spreads, preserves and related products	356	283	44.3
Confectionery, biscuits and snack foods	272	932	77.4
Special-diet, plant-based and special-purpose products	206	230	52.8
Infant formula and complementary foods	37	63	63.0
Oils, margarines and fat spreads	162	37	18.6
Preserved foods and ready meals	280	157	35.9
Sauces, condiments and seasonings	206	213	50.8
Frozen foods	95	105	52.5

Panel B. *Per-profile ascertainment (measurement sample) and fiber declaration (distal sample).*

Profile	n (measurement)	CNO, n (%)	MNS, n (%)	n (distal)	No fiber declaration, n (%)
P1 Sparse	1041	196 (18.8)	42 (4.0)	903	141 (15.6)
P2 Oxidation/color retention	845	184 (21.8)	66 (7.8)	742	143 (19.3)
P3 Raising/texture	803	132 (16.4)	24 (3.0)	677	154 (22.7)
P4 Coating/structure	518	158 (30.5)	88 (17.0)	447	107 (23.9)
P5 Carbonation/pressurization	176	103 (58.5)	0 (0.0)	173	1 (0.6)

Panel C. *Fiber non-declaration by food group (distal sample).*

Food group	n (distal)	No fiber declaration, n	%
Processed meat and packaged poultry products	137	133	97.1
Dairy products and milk-based beverages	301	65	21.6
Bakery, cereal and pasta products	442	54	12.2
Non-alcoholic beverages	328	17	5.2
Breakfast spreads, preserves and related products	282	28	9.9
Confectionery, biscuits and snack foods	879	78	8.9
Special-diet, plant-based and special-purpose products	124	5	4.0
Oils, margarines and fat spreads	31	28	90.3
Preserved foods and ready meals	149	67	45.0
Sauces, condiments and seasonings	194	18	9.3
Frozen foods	75	53	70.7

Panel D. *Food-group composition of the profiles: n (column %), measurement sample, modal assignment.*

Food group	P1 Sparse	P2 Oxidation/color retention	P3 Raising/texture	P4 Coating/structure	P5 Carbonation/pressurization
Processed meat and packaged poultry products	20 (1.9)	15 (1.8)	71 (8.8)	33 (6.4)	1 (0.6)
Dairy products and milk-based beverages	137 (13.2)	34 (4.0)	51 (6.4)	163 (31.5)	0 (0.0)
Bakery, cereal and pasta products	177 (17.0)	97 (11.5)	104 (13.0)	71 (13.7)	0 (0.0)
Non-alcoholic beverages	28 (2.7)	75 (8.9)	55 (6.8)	59 (11.4)	172 (97.7)
Breakfast spreads, preserves and related products	132 (12.7)	135 (16.0)	10 (1.2)	6 (1.2)	0 (0.0)
Confectionery, biscuits and snack foods	250 (24.0)	171 (20.2)	406 (50.6)	103 (19.9)	2 (1.1)
Special-diet, plant-based and special-purpose products	99 (9.5)	49 (5.8)	43 (5.4)	39 (7.5)	0 (0.0)
Infant formula and complementary foods	3 (0.3)	24 (2.8)	33 (4.1)	3 (0.6)	0 (0.0)
Oils, margarines and fat spreads	12 (1.2)	25 (3.0)	0 (0.0)	0 (0.0)	0 (0.0)
Preserved foods and ready meals	39 (3.7)	114 (13.5)	3 (0.4)	1 (0.2)	0 (0.0)
Sauces, condiments and seasonings	122 (11.7)	81 (9.6)	7 (0.9)	3 (0.6)	0 (0.0)
Frozen foods	22 (2.1)	25 (3.0)	20 (2.5)	37 (7.1)	1 (0.6)

Panel E. *FVL-indeterminate exclusions (n = 108) by profile and food group.*

Food group	P1 Sparse	P2 Oxidation/color retention	P3 Raising/texture	P4 Coating/structure
Bakery, cereal and pasta products	2	0	0	0
Non-alcoholic beverages	6	3	0	4
Special-diet, plant-based and special-purpose products	24	18	3	19
Preserved foods and ready meals	0	1	0	0
Frozen foods	3	14	7	4

Panel F. *Record-level quality control during inventory construction and revision. Potentially overlapping records were screened using normalized product-identification information, standardized declared ingredient lists, and package-image review; the assessment was not a comprehensive longitudinal barcode-based reformulation audit.*

Audit item	Result
Completed records	6526
Confirmed same-source duplicate entries excluded	2
Records excluded because ingredient lists were not transcribed	9
Final analytical inventory	6515
Additional confirmed duplicate entries requiring exclusion after revision-stage review	0

Panel G. *Unresolved additive declarations by food group (analytical inventory, n = 6515). Of the 28 CNO products outside the measurement sample, 25 had no exactly resolved INS entry and 3 carried exactly resolved entries only on the marker exclusion list.*

Food group	Inventory, n	CNO products, n (%)	CNO records, n	CNO products without a resolved INS entry, n	MNS products, n (records)
Processed meat and packaged poultry products	200	14 (7.0)	22	1	0 (0)
Dairy products and milk-based beverages	729	38 (5.2)	41	0	113 (121)
Bakery, cereal and pasta products	1232	250 (20.3)	589	4	7 (7)
Non-alcoholic beverages	720	149 (20.7)	286	2	0 (0)
Breakfast spreads, preserves and related products	639	8 (1.3)	8	0	3 (3)
Confectionery, biscuits and snack foods	1204	141 (11.7)	236	4	33 (33)
Special-diet, plant-based and special-purpose products	436	79 (18.1)	139	3	17 (17)
Infant formula and complementary foods	100	1 (1.0)	1	1	0 (0)
Oils, margarines and fat spreads	199	0 (0.0)	0	0	0 (0)
Preserved foods and ready meals	437	14 (3.2)	15	5	0 (0)
Sauces, condiments and seasonings	419	38 (9.1)	40	1	56 (57)
Frozen foods	200	69 (34.5)	246	4	0 (0)
Total	6515	801 (12.3)	1623	25	229 (238)

Panel H. *Measurement-eligible versus ineligible products in the analytical inventory (n = 6515; descriptive, no significance tests).*

Characteristic	Measurement-eligible (*n* = 3383)	Ineligible (*n* = 3132)
Ingredients per product, median (IQR)	13 (8–21)	2 (1–5)
Within Nutri-Score scope, %	97.4	98.8
Any class-name-only declaration, %	22.8	0.9
Any modified-starch-only declaration, %	6.5	0.3
No ingredient record classified as an additive, %	0.0	98.1
Ineligibility reason: no declaration resolved to an exact INS entry, n	–	3106
Ineligibility reason: only marker-list INS entries, no retained indicator, n	–	26
Food group, n (column %)		
Processed meat and packaged poultry products	140 (4.1)	60 (1.9)
Dairy products and milk-based beverages	385 (11.4)	344 (11.0)
Bakery, cereal and pasta products	449 (13.3)	783 (25.0)
Non-alcoholic beverages	389 (11.5)	331 (10.6)
Breakfast spreads, preserves and related products	283 (8.4)	356 (11.4)
Confectionery, biscuits and snack foods	932 (27.5)	272 (8.7)
Special-diet, plant-based and special-purpose products	230 (6.8)	206 (6.6)
Infant formula and complementary foods	63 (1.9)	37 (1.2)

Characteristic	Measurement-eligible (*n* = 3383)	Ineligible (*n* = 3132)
Oils, margarines and fat spreads	37 (1.1)	162 (5.2)
Preserved foods and ready meals	157 (4.6)	280 (8.9)
Sauces, condiments and seasonings	213 (6.3)	206 (6.6)
Frozen foods	105 (3.1)	95 (3.0)
Calculated Nutri-Score grade, n (column %); products with a grade, excluding water: 3050 and 2317		
Grade A	121 (4.0)	744 (32.1)
Grade B	110 (3.6)	383 (16.5)
Grade C	487 (16.0)	430 (18.6)
Grade D	966 (31.7)	461 (19.9)
Grade E	1366 (44.8)	299 (12.9)

Panel H compares measurement-eligible (n = 3383) and ineligible (n = 3132) products. Investigator-computed Nutri-Score grades exclude plain water and include only products within the algorithm scope with a derivable grade (n = 3050 and 2,317, respectively); among eligible products, these comprise the distal sample (n = 2942) and the 108 products excluded at the distal gate for indeterminate FVL content. Absence of a retained exact INS-linked indicator does not establish that a product is additive-free.

Supplementary Table S13. Sensitivity of the first-step variance correction to measurement-parameter draws. Proportional odds models used 100, 500, and 1000 nested draws; key partial proportional odds terms used 100. Percentage increases over conditional standard errors were calculated from unrounded values. Twenty-one boundary parameters were held fixed, which may understate uncertainty in highly separated cells.

Model	Draws	Term	Conditional SE	Corrected SE	Increase, %
PO	100	P2	0.1132	0.1134	0.130
PO	100	P3	0.1421	0.1424	0.259
PO	100	P4	0.1384	0.1389	0.338
PO	100	P5	0.5696	0.5697	0.019
PO	500	P2	0.1132	0.1134	0.133
PO	500	P3	0.1421	0.1424	0.213
PO	500	P4	0.1384	0.1388	0.321
PO	500	P5	0.5696	0.5702	0.106
PO	1000	P2	0.1132	0.1134	0.134
PO	1000	P3	0.1421	0.1424	0.237
PO	1000	P4	0.1384	0.1389	0.333
PO	1000	P5	0.5696	0.5700	0.082
PPO	100	P3, common	0.1418	0.1422	0.231
PPO	100	P2, ABCD|E	0.1361	0.1363	0.191
PPO	100	P4, A|BCDE	0.5918	0.5927	0.150
PPO	100	P4, AB|CDE	0.2676	0.2680	0.138

Supplementary Table S14. Profile 5 (carbonation/pressurization) associations with Nutri-Score grade. The profile is mapping-sensitive and is not substantively interpreted (Section 3.3); its estimates are omitted from Table 2 of the main text and are shown here for completeness. Model A, profiles only; Model B, plus functional-class count. Reference: P1 Sparse.

Cutpoint	Model A OR (95% CI)	Model B OR (95% CI)
B–E vs. A	14.03 (1.40–140.65)	10.80 (1.05–111.56)
C–E vs. A–B	1.14 (0.33–3.96)	0.88 (0.24–3.22)
D–E vs. A–C	1.29 (0.40–4.12)	0.99 (0.29–3.36)
E vs. A–D	3.13 (0.99–9.96)	2.41 (0.72–8.12)

Supplementary Table S15. Revision-stage nonsweetener-function restoration specification with class re-enumeration (26 indicators; every nonsweetener Codex class of the excluded marker substances restored; measurement sample *n* = 3383): class enumeration and half-sample stability (Panel A), matching to the primary profiles (Panel B), and distal analysis on the selected six-class solution (Panel C; distal sample *n* = 2942).

Panel A. *Class enumeration and stability.*

Classes	Log-likelihood	BIC	Entropy	Smallest class, n (%)	Boundary probabilities, n	Half-sample, Variant B	Half-sample, half-to-half
1	-45,452.0	91,115.4	1.000	3383 (100.0)	0	–	–
2	-39,133.9	78,698.6	0.916	1635 (48.3)	0	–	–
3	-36,195.9	73,041.9	0.952	906 (26.8)	4	50/50	50/50
4	-34,435.3	69,740.1	0.972	647 (19.1)	18	16/50	16/50
5	-32,751.8	66,592.5	0.979	176 (5.2)	28	19/50	26/50
6	-31,603.1	64,514.5	0.975	176 (5.2)	31	37/50	29/50
7	-30,616.3	62,760.3	0.981	176 (5.2)	52	3/50	34/50
8	-29,803.9	61,355.1	0.984	176 (5.2)	58	18/50	31/50
9	-29,265.6	60,497.9	0.982	176 (5.2)	68	–	–
10	-28,824.6	59,835.3	0.981	175 (5.2)	80	–	–
11	-28,416.9	59,239.3	0.977	74 (2.2)	100	–	–
12	-27,984.4	58,593.7	0.979	74 (2.2)	100	–	–

Panel B. *Matching to the primary profiles: Pearson correlation of conditional-probability vectors (modal agreement with the primary assignment).*

Primary profile	Selected six-class solution	Secondary variant, six classes (*n* = 3396)	Five-class solution (not selected)
P1 Sparse	0.927 (828 of 1041)	0.924 (824 of 1041)	0.754 (644 of 1041)
P2 Oxidation/color retention	0.999 (798 of 845)	0.999 (798 of 845)	0.999 (805 of 845)
P3 Raising/texture	0.981 (545 of 803)	0.981 (545 of 803)	0.936 (646 of 803)
P4 Coating/structure	0.874 (325 of 518)	0.874 (325 of 518)	0.784 (189 of 518)
P5 Carbonation/pressurization	0.974 (176 of 176)	0.974 (176 of 176)	0.974 (176 of 176)
Unmatched class	418 products	432 products (10 of the 13 added products)	–

Panel C. *Distal analysis on the six-class solution: odds ratios (95% CI) relative to the class matched to P1 Sparse.*

Class	Cutpoint	Partial proportional odds, Model A	With count (Model B)	Proportional odds, common OR	Holm-adjusted p (PO test)
P2 Oxidation/color retention (matched)	B–E vs. A	1.03 (0.61–1.75)	0.68 (0.37–1.26)	0.69 (0.54–0.89)	<0.0001
	C–E vs. A–B	1.34 (0.86–2.10)	0.88 (0.51–1.52)		
	D–E vs. A–C	1.05 (0.77–1.44)	0.67 (0.42–1.06)		
	E vs. A–D	0.45 (0.33–0.60)	0.28 (0.18–0.45)		
P3 Raising/texture (matched)	B–E vs. A	2.00 (0.87–4.60)	1.15 (0.46–2.89)	1.41 (1.03–1.95)	0.0061
	C–E vs. A–B	2.08 (1.08–4.02)	1.18 (0.55–2.56)		
	D–E vs. A–C	1.14 (0.76–1.71)	0.64 (0.36–1.13)		
	E vs. A–D	1.39 (0.98–1.97)	0.78 (0.46–1.33)		
P4 Coating/structure (matched)	B–E vs. A	6.95 (2.67–18.07)	3.32 (1.15–9.61)	1.14 (0.86–1.52)	0.0008
	C–E vs. A–B	1.85 (1.09–3.13)	0.88 (0.44–1.80)		
	D–E vs. A–C	1.41 (0.98–2.02)	0.67 (0.37–1.22)		
	E vs. A–D	0.91 (0.66–1.27)	0.43 (0.24–0.78)		
P5 Carbonation/pressurization (matched)	B–E vs. A	16.37 (1.62–165.49)	8.06 (0.76–85.31)	2.01 (0.65–6.21)	<0.0001
	C–E vs. A–B	1.40 (0.40–4.92)	0.68 (0.18–2.59)		
	D–E vs. A–C	1.38 (0.43–4.44)	0.66 (0.19–2.34)		
	E vs. A–D	2.67 (0.84–8.52)	1.29 (0.37–4.51)		
Unmatched intermediate class	B–E vs. A	4.39 (1.77–10.86)	2.82 (1.09–7.32)	1.12 (0.82–1.51)	0.0008
	C–E vs. A–B	4.12 (2.06–8.21)	2.58 (1.20–5.54)		
	D–E vs. A–C	1.66 (1.12–2.45)	1.06 (0.64–1.76)		
	E vs. A–D	0.72 (0.50–1.03)	0.47 (0.29–0.76)		

This revision-stage analysis was specified before execution, with class selection recorded before distal modeling; it was not prespecified. Enumeration used the primary settings (Variant B: 400 full-sample and 120 half-sample starts; half-to-half: 60 starts per half), without numerical cutoffs. Six classes were selected because three merged the interpreted profiles, four and five showed low recovery, six showed the highest Variant B recovery among the evaluated solutions with four or more classes and recovered counterparts for all primary profiles, and seven and eight showed a marked rise in boundary probabilities with discordant recovery. Matching correlations describe profile similarity, not preservation of modal assignments (P3: 545/803; P4: 325/518). The unmatched intermediate class and P5 were not interpreted. The global proportional odds test rejected proportionality (chi-square 133.7, df = 15, p < 0.001), and all profile terms were freed after Holm adjustment. The restored functional-class count (restricted cubic spline, knots 1, 8, and 14) improved fit (chi-square 9.51, df = 2, p = 0.009). Restoring flavor enhancer, the only nonsweetener class of several intense sweeteners, partly reintroduced sweetener information; the secondary variant with 13 additional marker-only products was assessed only at the measurement stage. Distal models used a food-subgroup random intercept and fixed-parameter two-step estimation.

Supplementary Table S16. Brand-level cluster bootstrap and product-level bootstrap of four selected terms of the adopted partial proportional odds Model A (distal sample, *n* = 2942).

Term	OR	Model 95% CI	Model SE	Product SE	Brand SE	Brand/product SE ratio	Brand percentile 95% CI
P3 Raising/texture, common	1.99	1.51–2.63	0.142	0.154	0.189	1.23	1.47–3.03
P2 Oxidation/color retention, E vs. A–D	0.49	0.37–0.63	0.136	0.145	0.196	1.36	0.33–0.67
P4 Coating/structure, B–E vs. A (sparse; see note)	10.87	3.41–34.68	0.592	2.082	1.885	0.91	4.50–7199.01
P4 Coating/structure, C–E vs. A–B	1.95	1.16–3.30	0.268	0.277	0.377	1.36	0.99–4.63

This revision-stage analysis was specified before execution but was not prespecified. Model, model-based; Product, product-level bootstrap; Brand, brand-cluster bootstrap. Normalized brand names defined 654 clusters (largest, 131 products); records without a brand formed singleton clusters, and brands were not assumed to identify manufacturers. No brand names are reported. Each of 200 brand-cluster and 200 product-level resamples refitted Model A with fixed measurement probabilities and partial proportional odds structure, re-estimated class proportions, and reindexed food subgroups; no replicate was excluded for non-convergence or invalid cutpoints. Bootstrap standard errors are on the log-odds scale, and percentile intervals are approximate. The product-level bootstrap separates the effect of clustering from that of using a bootstrap. For the sparse P4 B–E versus A contrast, 6 brand-cluster and 10 product-level replicates yielded ORs above 1000; the corresponding bootstrap standard errors were therefore unreliable.

Supplementary Figure S1. Common and cutpoint-specific associations for all five latent profiles, including the mapping-sensitive Profile 5 (complete version of main-text Figure 3). Model A, filled circles; Model B, open diamonds. Profile 5 is shown in gray and is not substantively interpreted.

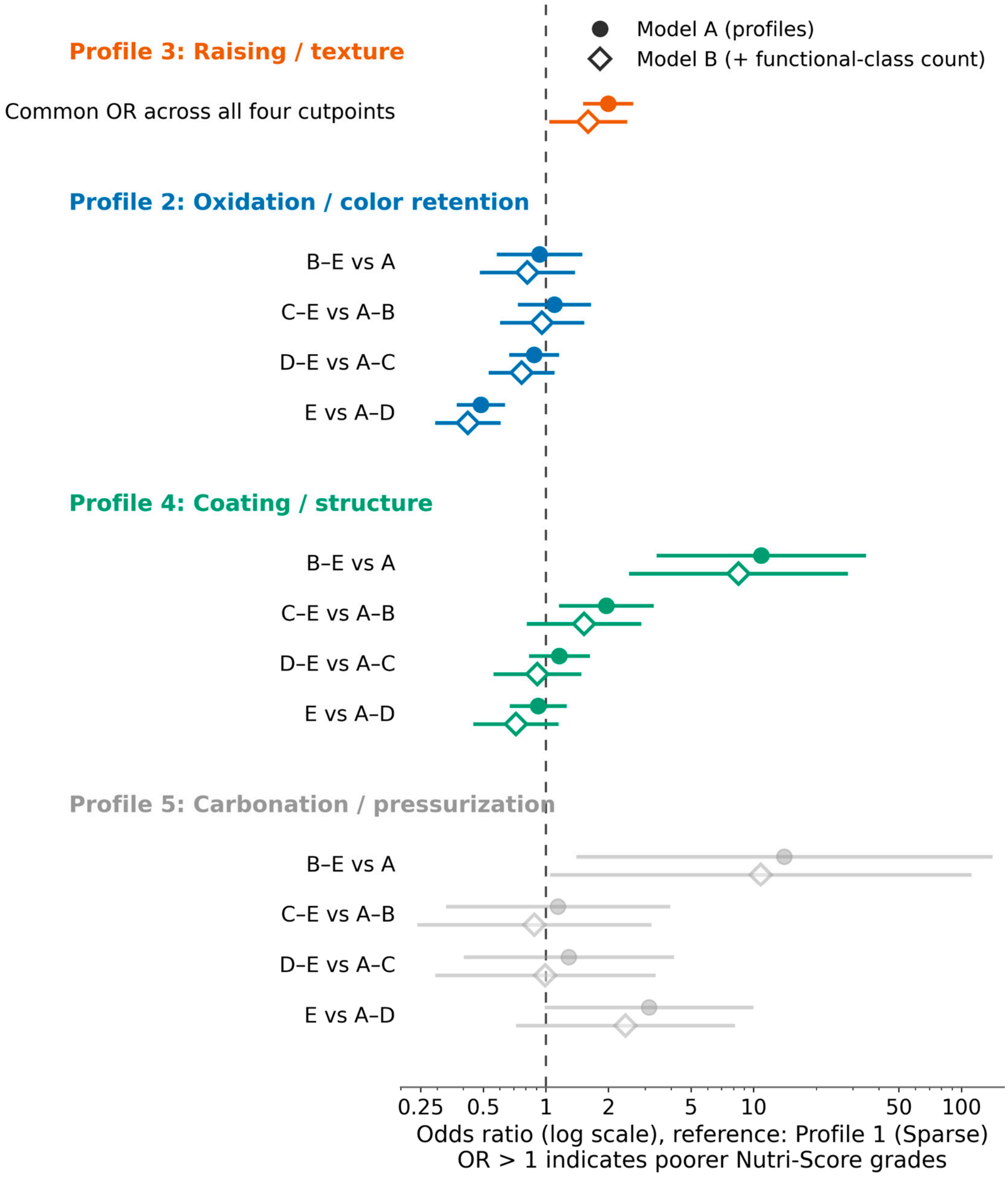

Supplementary Figure S2. Proportional-odds sensitivity version of the observed and model-standardized Nutri-Score grade distributions (main-text Figure 4), with panel (b) computed from the proportional odds form of Model A. Main-text Figure 4 computes panel (b) from the adopted partial proportional odds model; the largest absolute cell-wise difference between the two versions is 0.156 (Profile 5, grade D).

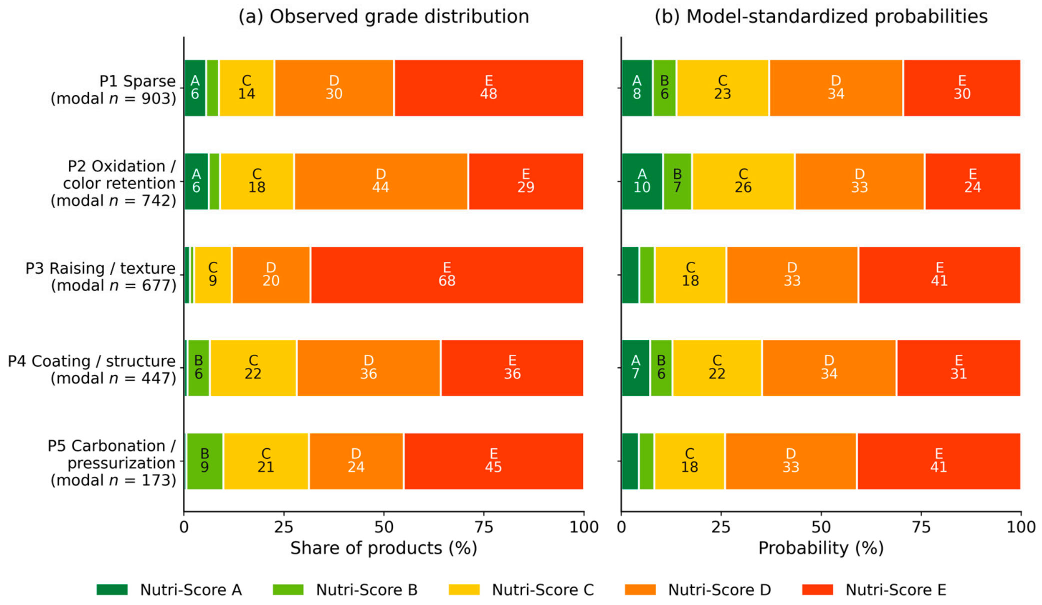

Supplement: Supplementary file 1 [file foods-15-03838-s001.zip › foods-4608133-supplementary.pdf]
